# Autism-Relevant Social Abnormalities and Cognitive Deficits in *Engrailed-2* Knockout Mice

**DOI:** 10.1371/journal.pone.0040914

**Published:** 2012-07-19

**Authors:** Jennifer Brielmaier, Paul G. Matteson, Jill L. Silverman, Julia M. Senerth, Samantha Kelly, Matthieu Genestine, James H. Millonig, Emanuel DiCicco-Bloom, Jacqueline N. Crawley

**Affiliations:** 1 Laboratory of Behavioral Neuroscience, National Institute of Mental Health, Bethesda, Maryland, United States of America; 2 Center for Advanced Biotechnology and Medicine, University of Medicine and Dentistry of New Jersey-Robert Wood Johnson Medical School, Piscataway, New Jersey, United States of America; 3 Neuroscience and Cell Biology, University of Medicine and Dentistry of New Jersey-Robert Wood Johnson Medical School, Piscataway, New Jersey, United States of America; 4 Pediatrics, University of Medicine and Dentistry of New Jersey-Robert Wood Johnson Medical School, New Brunswick, New Jersey, United States of America; Université Pierre et Marie Curie, France

## Abstract

*ENGRAILED 2 (En2)*, a homeobox transcription factor, functions as a patterning gene in the early development and connectivity of rodent hindbrain and cerebellum, and regulates neurogenesis and development of monoaminergic pathways. To further understand the neurobiological functions of *En2*, we conducted neuroanatomical expression profiling of *En2* wildtype mice. RTQPCR assays demonstrated that *En2* is expressed in adult brain structures including the somatosensory cortex, hippocampus, striatum, thalamus, hypothalamus and brainstem. Human genetic studies indicate that *EN2* is associated with autism. To determine the consequences of *En2* mutations on mouse behaviors, including outcomes potentially relevant to autism, we conducted comprehensive phenotyping of social, communication, repetitive, and cognitive behaviors. *En2* null mutants exhibited robust deficits in reciprocal social interactions as juveniles and adults, and absence of sociability in adults, replicated in two independent cohorts. Fear conditioning and water maze learning were impaired in *En2* null mutants. High immobility in the forced swim test, reduced prepulse inhibition, mild motor coordination impairments and reduced grip strength were detected in *En2* null mutants. No genotype differences were found on measures of ultrasonic vocalizations in social contexts, and no stereotyped or repetitive behaviors were observed. Developmental milestones, general health, olfactory abilities, exploratory locomotor activity, anxiety-like behaviors and pain responses did not differ across genotypes, indicating that the behavioral abnormalities detected in *En2* null mutants were not attributable to physical or procedural confounds. Our findings provide new insight into the role of *En2* in complex behaviors and suggest that disturbances in *En2* signaling may contribute to neuropsychiatric disorders marked by social and cognitive deficits, including autism spectrum disorders.

## Introduction

Mutations in genes that control early neurodevelopmental processes impact complex behaviors in mice, including social behaviors [1]–[4], cognitive abilities [3], , anxiety- and depression-related behaviors [2], [7], [9], [12], [13], and motor functions [5], [6], [14]. There is compelling evidence that disturbances in neurodevelopment underlie psychiatric disorders such as autism spectrum disorders (ASD) and schizophrenia [15]–[18]. Comprehensive phenotyping of mice with targeted mutations in neurodevelopmental genes could shed light on the mechanisms that contribute to the social impairments, cognitive deficits and other behavioral abnormalities that characterize these disorders.

*Engrailed-2* is a homeobox transcription factor that coordinates multiple aspects of CNS development [19], [20] and is upregulated during neural differentiation [21]. In the developing mouse brain, *En2* restricts the fate of progenitor cells to a midbrain or hindbrain lineage [22], [23] and regulates cerebellar patterning and connectivity [24]–[30]. Animal studies have also demonstrated that *En2* coordinates the development and maintenance of monoaminergic neurons [31]–[35] and retinal-tectal axon guidance [36], [37]. *En2* developmental studies have focused on the mid-hindbrain because the gene is expressed at highest levels in these structures [20], [22], [23], [38], and in adulthood, *En2* is expressed primarily in mature cerebellar granule cells [23], [38]. However, recent RTQPCR analysis indicated that *En2* is transcribed at lower levels in the hippocampus and cortex, and is associated with kainic acid induced seizures and possibly excitatory/inhibitory circuit imbalance [39].

Mice with a deletion in *En2* display multiple neuroanatomical abnormalities including cerebellar hypoplasia, reduced Purkinje cell numbers, disruptions in cerebellar patterning and foliation, reduced hippocampal weight, increased dentate gyrus cell turnover and an anterior shift in the position of the amygdala [25], [26], [28], [40]–[42]. Neurochemical investigations revealed that *En2* null mutant mice display perturbations in monoamine neurotransmitter pathways. *En2* null mutants exhibit reduced levels of tyrosine hydroxylase, norepinephrine and/or serotonin in the hippocampus and cerebral cortex with increased levels of these transmitters in the cerebellum [42]–[44].

Previous human genetic studies have demonstrated that *EN2* is significantly associated with autism spectrum disorders (ASD). The common alleles (underlined) of two intronic *EN2* SNPs, *rs1861972* (A/G) and *rs1861973* (C/T), are inherited more frequently in affected individuals while the G–T haplotype is overrepresented in unaffected siblings [43], [44]. These results were initially observed in 167 families and then replicated in two additional datasets (518 families; P = 00000035). The A–C haplotype was also recently found to be functional and increases gene expression [45]. Six other groups have demonstrated association for *EN2* with ASD [46]–[51], supporting the interpretation that *EN2* is an ASD susceptibility gene.

To further understand the functions of *En2*, we conducted comprehensive behavioral phenotyping of mice with a deletion in *En2*. Although the human A–C allele changes protein expression in the opposite direction to the mouse knockout, we reasoned that explicating the basic role of *En2* in mediating mouse behaviors would shed light on our understanding of the functions of this developmental gene. Considering the consequences of other homeobox genes on cognitive abilities, we evaluated *En2* mutant mice on three cognitive tasks. Given the association between *EN2* mutations and autism, we evaluated a wide range of behavioral phenotypes relevant to the diagnostic and associated symptoms of ASD, along with control measures. Male and female littermates of all three genotypes (*En2*+/+, +/− and −/−) were tested across developmental ages in two large independent cohorts of mice. To begin to identify possible neuroanatomical substrates for these behavioral phenotypes, we employed RTQPCR to determine the distribution of *En2* expression throughout the +/+ adult mouse brain. Our findings, which confirm and extend previous reports, indicate the importance of *En2* in regulation of social behaviors, cognitive abilities and motor functions in mice, the disruption of which may lead to behavioral phenotypes relevant to autism and related neuropsychiatric disorders.

## Materials and Methods

### Ethics Statement

All experimental protocols were conducted in strict compliance with the National Institutes of Health Guidelines for the Care and Use of Laboratory Animals and approved by the NIMH Animal Care and Use Committee and the UMDNJ Institutional Animal Care and Use Committee.

### Mice

*En2^ tm1Alj/tm1Alj^* (*En2*−/− mice), generated on a 129S2/SvPas background as previously described [20], [25], [26], were purchased from The Jackson Laboratories (Bar Harbor, ME) and delivered to the University of Medicine and Dentistry of New Jersey-Robert Wood Johnson Medical School (UMDNJ-RWJMS) in Piscataway, NJ. *En2* heterozygous offspring on the B6/Pas hybrid genetic background were intercrossed to non-littermates to maintain the line. The mice were maintained on a 12∶12 light:dark cycle as approved by the RWJMS IACUC. *En2* heterozygous breeding pairs were imported from UMDNJ to the National Institute of Mental Health (NIMH) in Bethesda, MD for behavioral testing. Heterozygotes were bred in a conventional mouse vivarium using harem breeding trios. Pups were kept with the dam until weaning at postnatal day (pnd) 21. After weaning, juveniles were housed by sex in groups of two to four. All experiments were conducted using *En2* wildtype (+/+), heterozygote (+/−) and null mutant (−/−) male and female littermates. Mice were housed in standard plastic cages in a colony room maintained at approximately 20°C, with *ad libitum* access to food and water. The colony room was maintained on a 12∶12 light:dark cycle with lights on at 06∶00 hours.

### Genotyping

Mice bred in Bethesda were genotyped by PCR analysis of tail DNA using standard PCR methods. Briefly, 0.5–1 cm tail snips were digested using the Promega Wizard SV Genomic DNA Purification System (Promega, Madison, WI). The following primers were utilized in the PCR reaction: GTTCACAGTCCTGTGAAATGCAGC, a sequence common to both *En2*+/+ and En2−/− mice; (2) ACCAACAGGTACCTGACAGAGC, a sequence specific for the *En2*+/+ homeobox; and (3) CTTGGGTGGAAGGGCTATTC, a sequence in the neomycin gene in the *En2*−/− mutation. These primers amplify a 600-bp band in *En2*+/+ mice, a 950-bp band in *En2*−/− mice, and one band of each size in *En2*+/− mice.

### RTQPCR

First strand cDNA was generated using 1 µg of RNA and High-Capacity cDNA Reverse Transcription Kit (Applied Biosystems, Foster City, CA) following manufacturer’s instructions and an *En2* primer (GAAGATGATTCCAACTCGCTCT). Quantitative PCR was conducted using one twentieth of total cDNA and Taqman® probe sets for mouse *En2* (Mm00438710_m1, fluorescent dye FAM labeled) and *GAPDH* internal control (4352339E, fluorescent dye VIC labeled) on ABI7900HT (Applied Biosystems). *En2* level was normalized to endogenous *Gapdh* level by subtracting *Gapdh* Ct from *En2* Ct (▵Ct). The average of the normalized Ct (▵Ct) values was obtained from three replicates of qRT-PCR reaction.

### Behavioral Tests

Behavioral experiments were conducted between 10∶00 and 16∶00 in dedicated testing rooms, using methods previously described [52]–[55]. To evaluate the replicability of behavioral phenotypes detected, most tasks were repeated using a second cohort of mice. Identification was done by paw tattooing at age 2–4 days. All behavioral ratings were conducted by investigators who were blind to the genotype of the subject mice. To ensure that investigators were unaware of the genotype during the test session, tattoo markings were recorded in the datasheet only after the end of the test session, for each subject mouse. In cases where scoring was conducted from videotapes, the video was assigned a code number, and the genotype identification number of the subject mouse was attached to the data after completion of the experiment.

Order of testing was as follows: (1) developmental milestones across postnatal days 2–14 in one cohort; pup ultrasonic vocalizations on postnatal days 4, 6, 8 and 11 in a separate cohort; (2) juvenile reciprocal social interactions at age 20–22 days; (3) elevated plus-maze and light *↔* dark exploration tests for anxiety-related behaviors at age 6–7 weeks; (4) open field locomotion and rotarod motor coordination and balance at age 8–9 weeks; (5) adult 3-chambered social approach at age 8–10 weeks; (6) general health, neurological reflexes, pain sensitivity and grip strength at age 9–11 weeks; (7) novel object recognition memory test at 10–11 weeks; (8) adult male-female social interactions at age 9–12 weeks; (9) self-grooming at age 12–13 weeks; (10) olfactory habituation/dishabituation at age 13–14 weeks; (11) acoustic startle and prepulse inhibition at age 14–16 weeks; (12) tail suspension and forced swim tests at age 15–16 weeks; (13) fear conditioning at age 16–18 weeks; and (14) Morris water maze at age 18–20 weeks. Males and females were used in approximately equal proportions for each experiment.

### Developmental Milestones

Developmental milestones were assayed in *En2* pups using a modified Fox battery [56], [57] as previously described [58]. Every other day from pnd 2 to 14, body temperature and somatic growth parameters including body weight, body length, day of eyelid opening and pinnae detachment were measured. Reflexes and responses including negative geotaxis, vertical screen climbing, righting reflex and auditory startle were assayed on the same days, as previously described [58]. Body weight was measured to the nearest 0.1 g and body temperature to the nearest 0.01°C. Latency to display the righting reflex was measured in seconds using a stopwatch. Other somatic and behavioral variables were rated semi-quantitatively using the following scoring system: 0 =  no response or occurrence of the event, 1 =  slight response or occurrence of the event, 2 =  incomplete or ambiguous response or occurrence of the event, and 3 =  complete, unambiguous response or occurrence of the event. Investigators were trained until the inter-observer reliability was greater than 95%. The absence of a milestone was scored as zero if the mouse did not exhibit the behavior within 60 seconds.

### Pup Ultrasonic Vocalizations

Ultrasonic vocalizations (USVs) were recorded from *En2* pups separated from the mother and nest as previously described [53], [58], [59]. Litters tested for ultrasonic vocalizations were not used for developmental milestones testing, to avoid potential confounds from using previously handled animals. Measurements of USVs were taken on postnatal days 4, 6, 8 and 11. On each day of testing, the pup was removed from the home cage and placed into an empty glass container (5×10 cm) situated inside a sound-attenuating styrofoam box. USVs were recorded over a 3 minute recording session. At the end of the recording session, each pup was weighed and its body temperature measured. The temperature of the room was maintained at 23±1°C.

Ultrasonic calls were recorded in a sound-attenuating environmental chamber using an ultrasound microphone (Avisoft UltraSoundGate condenser microphone capsule CM16, Avisoft Bioacoustics, Berlin, Germany) sensitive to frequencies of 10–180 kHz. The microphone was placed through a hole in the middle of the cover of a styrofoam sound-attenuating box, about 20 cm above the pup, and connected to a PC installed with Avisoft Recorder software (version 3.2, Avisoft Bioacoustics, Berlin, Germany). The microphone sampling frequency was set to 205 kHz, and the resolution set to 16 bits. For acoustical analysis,. WAV files containing the USV recordings were transferred to Avisoft SASLab Pro software (version 4.40) and a fast Fourier transformation (FFT) was conducted. Spectrograms were generated with an FFT-length of 512 points and a time window overlap of 75% (100% Frame, Hamming window). The spectrogram was produced at a frequency resolution of 488 Hz and a time resolution of 1 ms. A lower cut-off frequency of 15 kHz was used to reduce background noise outside the relevant frequency band to 0 dB. Call detection was provided by an automatic threshold-based algorithm and a hold-time mechanism (hold time: 0.01 seconds). An experienced user checked the accuracy of call detection, and obtained greater than 99% concordance between automated and observational detection. Parameters analyzed for each test day included total number of calls, mean duration of calls, and mean call frequency and amplitude.

### Juvenile Reciprocal Social Interactions

Multiple parameters of social interactions were scored in freely moving pairs of juvenile mice aged 20–22 days as previously described [55], [60]–[63]. Subjects were individually housed in standard mouse cages for 1 hour prior to the test session. Testing was conducted in the Noldus PhenoTyper Observer 3000 chamber (30×30×30 cm, Noldus Information Technology, Leesburg, VA), with a thin layer of bedding covering the floor. The *En2* subject mouse was placed into the arena with an age- and sex-matched juvenile C57BL6/J (B6) partner mouse. B6 were chosen for the partners because this strain exhibits the high levels of social behaviors that characterize most inbred strains of mice, and B6 are neither unusually high nor unusually low on most behavior traits. Interactions were recorded for 10 minutes using a top-mounted CCTV camera (Security Cameras Direct, Luling, TX). Behaviors were subsequently scored from videotapes by a highly trained observer uninformed of genotype using Noldus Observer 8.0 XT software (Noldus Information Technology, Leesburg, VA). Parameters of interest were chosen from the literature and from our previous studies [61], [62], [64], [65]. Parameters scored included approaching the partner from the front, nose-to-nose sniffing and anogenital sniffing, grooming the partner, following the partner, pushing underneath the partner’s body or crawling over or under the partner (combined as a single parameter termed “push-crawl”). Bouts of arena exploration were scored as a control for general locomotor activity. Bouts of self-grooming were also scored.

### Elevated Plus-maze

The elevated plus-maze test for anxiety-like behavior was performed as previously described [66]–[68]. The apparatus (San Diego Instruments, San Diego, CA) was comprised of two open arms (30×5×5 cm) and two closed arms (30×5×15 cm) that extended from a common central platform (5×5 cm). Each mouse was individually placed in the center facing an open arm and allowed to freely explore the apparatus for 5 minutes. The illumination on the open arms was approximately 30 lux. The 5 minute session was recorded using a CCTV camera mounted overhead approximately 1 m from the plus-maze, for subsequent scoring of behavior. The apparatus was cleaned with 70% ethanol and water between subjects. Time spent in the open arms and numbers of open and closed arm entries were scored by a trained observer using Noldus Observer 8.0 XT software (Noldus Information Technology, Leesburg, VA). An open or closed arm entry was defined as all four paws in an arm. The number of open and closed arm entries was combined to yield a measure of total entries, which reflected general exploratory activity during the 5 minute test.

### Light ↔ Dark Exploration

The light ↔ dark exploration test for anxiety-like behavior was conducted as previously described [67], [69]. The apparatus consisted of a Plexiglas cage (45×26×28 cm) separated into two compartments by a partition, which had a small opening (10×5 cm) at floor level. The larger compartment (29.5×26×28 cm) was open on top, transparent, and illuminated by overhead fluorescent ceiling lights (350 lx). The smaller compartment (16.5×26×28 cm) was closed on top and painted black. The partition between compartments contained embedded photocells that detected beam breaks as the subject mouse moved between the light and dark compartments and was connected to a PC equipped with dedicated software (equipment and software built by George Dold and coworkers, Research Services Branch, NIH, Bethesda, MD). Mice were individually placed in the center of the light compartment, facing away from the partition, and allowed to freely explore the apparatus for 10 minutes. The number of transitions between the light and dark compartments, total time spent in each compartment, and the latency to the first entry from the light compartment to the dark compartment, were automatically recorded by the photocells embedded in the partition. Data from the beam breaks were automatically analyzed by the software.

### Open Field Activity

General exploratory activity in a novel open field was assessed using the automated VersaMax Animal Activity Monitoring System (AccuScan Instruments, Columbus, OH) as previously described [53], [55], [66]–[68]. The open field was a square arena (40×40×30.5 cm) equipped with photocell beams for automatic detection of activity. Mice were placed in the center of the open field and left to freely explore for a 30 minute test session. The number of horizontal and vertical beam breaks was taken as a measure of horizontal and vertical activity, respectively. Total distance traveled and time spent in the central 20×20 cm area of the open field were automatically recorded by the VersaMax system. Test chambers were cleaned with 70% ethanol between subjects.

### Rotarod

Motor coordination, balance and motor learning were assessed using an accelerating rotarod (Ugo Basile, Schwenksville, PA) as previously described [5], [53], [55], [63]. Mice were placed on a cylinder that slowly accelerated from 4 to 40 revolutions per minute over a 5-minute (300-second) test session. Two cohorts of mice were tested, each for a total of six trials. Mice tested in Cohort 1 were given two trials per day over three consecutive days, with an intertrial interval of 60 minutes. Mice tested in Cohort 2 were given three trials per day over two consecutive days, with an intertrial interval of 30 minutes. Latency to fall from the rotating rod was recorded with a maximum trial length of 300 seconds. A group of C57BL6/J mice were tested within the same experiment as *En2* mice from Cohort 1, to allow comparison of baseline motor performance.

### Sociability

Adult sociability was tested in our automated three-chambered social approach apparatus using methods previously described [55], [61], [70]–[72]. The apparatus was a rectangular box made of clear polycarbonate, divided into a center chamber and two side chambers. Retractable doors built into the two dividing walls allowed access to the side chambers. Number of entries and time spent in each of the three chambers were detected by photocells embedded in the doorways and automatically recorded by the software. Equipment and the Labview software program were designed and built by George Dold and coworkers, Section on Instrumentation, NIH, Bethesda, MD. A top mounted CCTV camera (Security Cameras Direct, Luling, TX) was positioned over the box to videotape the session. Time spent sniffing the novel mouse and time spent sniffing the novel object were subsequently scored from the videos by investigators who were uninformed of the genotype of the subject mouse.

The subject mouse was acclimated to the apparatus before sociability testing, beginning with a 10 minute habituation session in the empty center chamber, followed by a 10 minute habituation to all three empty chambers. The second habituation session served to confirm a lack of innate side chamber preference. The subject was then briefly confined to the center chamber while a novel object (inverted wire pencil cup, Galaxy, Kitchen Plus, http://www.kitchen-plus.com) was placed in one side chamber and a novel mouse contained inside an identical inverted wire cup was placed in the other side chamber. Mice used as the novel mouse stimuli were age- and sex-matched 129S1/SvImJ mice obtained from The Jackson Laboratory (Bar Harbor, ME), a strain that is relatively inactive. After both stimuli were positioned, the two side doors were lifted and the subject mouse was allowed access to all three chambers for 10 minutes. Time spent in each chamber and number of entries was automatically recorded by the software. Number of entries served as a within-task control for levels of general exploratory locomotion. Cumulative time spent sniffing the novel mouse and novel object were later scored by a trained observer uninformed of genotype. The apparatus was cleaned with 70% ethanol and water between subjects. At the end of each testing day, the boxes were thoroughly washed with soap and warm water and air dried.

### General Health, Neurological Reflexes and Pain Sensitivity

The general health of adult *En2* mice was assessed using methods previously described [55], [63], [68], [73]. Empty cage behaviors were scored by placing the mouse into a clean, empty cage and noting incidents of transfer freezing, wild running, stereotypies, and excessive exploration levels. General health evaluation included assessment of body weight, the condition of the fur and whiskers, skin color, limb tone and body tone. Neurological reflexes tested included whisker twitch, pinna twitch, eyeblink response, auditory startle, righting reflex, forepaw reaching and trunk curl. Behavioral reactivity was measured using tests assessing responsiveness to petting by the investigator, intensity of a dowel biting response and degree of struggling and vocalization during handling. Responsiveness to painful stimuli was assessed using the hot plate and tail flick tests as previously described [53], [68], [74]. For the hot plate test, the mouse was placed on the surface of a hot plate apparatus (Columbus Instruments, Columbus, OH) maintained at 55°C. Latency to the first paw lick, jump or vocalization was measured by an observer uninformed of genotype. A maximum cut-off latency of 30 seconds was used to prevent the risk of tissue damage to the paws. For the tail flick test, mice were gently restrained with the tail lying in the groove of a tail flick apparatus (Columbus Instruments). Thermal stimulation of the tail was provided by application of an intense photobeam. The latency for the mouse to move its tail out of the path of the beam was timed automatically by the apparatus. A maximum cut-off latency of 10 seconds was used to prevent the risk of tissue damage.

### Grip Strength

Forelimb grip strength was measured as an indicator of neuromuscular function as previously described [53], [75]. Mice were raised toward a grip strength meter (Columbus Instruments, Columbus, OH), positioned horizontally and allowed to grasp the pull bar of the apparatus using only their forepaws. Mice were slowly pulled by the base of the tail, away from the bar at a horizontal plane, until the forepaws released from the bar. The force applied to the bar at the moment the grasp was released was recorded as the peak tension. The test was repeated 3 consecutive times within the same session. The mean score of all 3 trials was used for data analysis.

### Male-Female Social Interactions

Male-female social interactions were evaluated in a 5-minute test session as previously described [76], [77]. Each of the *En2*+/+, +/− and −/− subject mice, aged 9–12 weeks, was paired with a different unfamiliar estrus B6 female. Both the subject mice and the female partner mice were group-housed. The test session was conducted in a clean cage with clean bedding, representing a novel situation for both the male subject and the female partner. A digital closed-circuit television camera (Panasonic, Secaucus, NJ) was positioned horizontally 30 cm in front of the cage.

Ultrasonic calls were recorded in a sound-attenuating chamber using an ultrasound microphone as previously described for recording of pup ultrasonic vocalizations. The microphone was mounted 20 cm above the test cage and the chamber was illuminated by a red light. Procedures for acoustical analysis and call detection were identical to those used for analysis of pup ultrasonic vocalizations.

Digital videos recorded during the test session were subsequently scored using Noldus Observer software (Noldus Information Technology, Leesburg, VA, USA) as previously described [76], [77]. Parameters scored included nose-to-nose sniffing, nose-to-anogenital sniffing, body sniffing and bouts of exploration of the test cage.

### Novel Object Recognition

The novel object recognition test was conducted in the open field arena using methods previously described [63], [78]. The experiment took place over two days and consisted of two habituation sessions, a 10 minute object familiarization session, and a 5 minute object recognition test. On day 1, each subject was habituated to a clean empty open field arena for 30 minutes. Twenty-four hours later, each mouse was returned to the open field for a second habituation phase which lasted 10 minutes. The mouse was then removed from the open field and placed in a clean temporary holding cage for approximately 2 minutes, during which time two identical objects were placed in the arena. Each subject was returned to the open field in which it had been habituated, and allowed to freely explore the objects for 10 minutes. After the object familiarization session, subjects were returned to their holding cages, which were transferred from the testing room to a nearby holding area. The open field was cleaned with 70% ethanol and let dry. One clean familiar object and one clean novel object were placed in the arena, where the two identical objects had been located during the familiarization phase. Thirty minutes after the familiarization session, each subject was returned to its open field for a 5 minute object recognition test, during which time it was allowed to freely explore the familiar object and the novel object. The familiarization session and recognition test were videotaped and subsequently scored by a highly trained investigator uninformed of genotype. Object investigation was defined as time spent sniffing the object when the nose was in contact with the object or within <2 cm from the object. Recognition memory was defined as spending significantly more time sniffing the novel object than the familiar object. Total time spent sniffing both objects was used as a measure of general exploration. Time spent sniffing two identical objects during the familiarization phase confirmed the lack of an innate side bias.

### Self-grooming

Mice were assessed for spontaneous self-grooming behaviors as previously described [53], [61], [62], [79]. Each mouse was placed individually into a clean standard mouse cage (46×23.5×20 cm) under dim light (25–30 lx). After a 10 minute habituation period, a highly trained observer who remained blind to genotype scored cumulative time spent grooming any region of the body over a 10 minute test session. The observer sat approximately 2 m from the test cage and scored time spent self-grooming with a silenced stopwatch.

### Olfactory Habituation/Dishabituation

Olfactory abilities were assessed using the olfactory habituation/dishabituation assay as previously described [55], [80], [81]. Prior to the start of testing, each mouse was placed into a clean standard cage containing fresh bedding and a plain cotton swab tip (MediChoice, Owens & Minor, Mechanicsville, VA) suspended from the cage lid. After a 45 minute acclimation period, olfactory testing began. Subjects were tested for time spent sniffing cotton swab tips saturated with familiar and unfamiliar odors, with and without social valence. Sequences of three identical swab tips assayed habituation to the same odor. A different odor presented on the swab tip assayed dishabituation, i.e. recognition that an odor is new. Swab tips were dipped in (1) distilled water, (2) almond extract (McCormick, Hunt Valley, MD; 1∶100 dilution) and (3) banana flavoring (McCormick, Hunt Valley, MD; 1∶100 dilution) to represent a range of non-social odors. Swabs were wiped across the bottom surface of a plastic cage that contained (4) sex-matched unfamiliar mice of a different strain, 129S1/SvImJ, and (5) sex-matched unfamiliar mice of another different strain, C57BL6/J, to represent two distinct social odors. Each swab was presented for a 2 minute period, immediately following the last swab presentation, for a total session length of approximately 30 minutes per mouse. Order of presentation of non-social and social odors was counterbalanced within each genotype.

### Acoustic Startle Threshold and Prepulse Inhibition

Acoustic startle threshold and prepulse inhibition (PPI) were measured using the SR-Lab System (San Diego Instruments, San Diego, CA) as previously described [53], [82], [83]. Each test session began by placing the mouse in the Plexiglas cylinder for a 5 minute acclimation period. A background noise level of 70 dB was maintained over the duration of the test sessions. Acoustic startle testing occurred over an 8 minute session. Mice were presented with each of six trial types across six discrete blocks of trials for a total of 36 trials. One trial type measured the response to no stimulus (baseline movement). The other five trial types measured the response to a 40 millisecond startle stimulus of 80, 90, 100, 110 or 120 dB. The six trial types were presented in pseudorandom order such that each trial type was presented once within a block of seven trials. PPI testing occurred over a 10.5 minute trial. Mice were presented with each of seven trial types across six discrete blocks of trials for a total of 42 trials. One trial type measured the response to no stimulus, and another to a 40 millisecond 110 dB startle stimulus. The other five trial types were acoustic prepulse plus acoustic startle stimulus trials. The 20 millisecond prepulse stimuli were sounds of 74, 78, 82, 86, or 92 dB, presented 100 milliseconds before the onset of the 110 dB startle stimulus. The seven trial types were presented in pseudorandom order such that each trial type was presented once within a block of seven trials. The intertrial interval was 10–20 seconds. For both acoustic startle and PPI testing, startle amplitude was measured every 1 milliseconds over a 65 ms period beginning at the onset of the startle stimulus. The maximum startle amplitude over this sampling period was taken as the dependent variable.

### Tail Suspension Test

The tail suspension test was conducted as previously described [52], [84]–[86]. Mice were securely fastened by taping the distal end of the tail to the edge of a metallic shelf, and suspended in a visually isolated area. A CCTV camera (Security Cameras Direct, Luling, TX) placed approximately 1 m in front of the shelf recorded each session for subsequent scoring of time spent immobile. The presence or absence of immobility, defined as the absence of limb movement, was sampled every 5 seconds over a 6 minute test session by a highly trained observer who remained blind to genotype. The shelf was cleaned with 70% ethanol between subjects.

### Forced Swim Test

The Porsolt forced swim test was conducted as previously described [86]–[88]. Mice were gently placed in a transparent Plexiglas cylinder (20 cm in diameter) filled to a depth of 15 cm with tap water (24+1°C). A CCTV camera (Security Cameras Direct, Luling, TX) placed 30–40 cm in front of the cylinder recorded each session for subsequent scoring of time spent immobile. The presence or absence of immobility, defined as the cessation of limb movements except minor movement necessary to keep the mouse afloat, was sampled every 5 seconds during the last 4 minutes of a 6 minute test session by a highly trained observer blind to genotype.

### Contextual and Cued Fear Conditioning

Standard delay contextual and cued fear conditioning were conducted as previously described [53], [89]. The conditioning chamber (32×25×23 cm, Med Associates, St. Albans, VT) was interfaced to a PC installed with VideoFreeze software (version 1.12.0.0, Med Associates) and enclosed in a sound-attenuating cubicle (64×76×42 cm, Med Associates). Training consisted of a 2 minute acclimation period followed by three tone-shock (CS–US) pairings (80 dB tone, duration 30 seconds; 0.5 mA footshock, duration 1 second; intershock interval 90 seconds) and a 2.5 minute period during which no stimuli were presented. Cumulative time spent freezing before and after the CS–US pairings was quantified by the VideoFreeze software. A 5 minute test of contextual fear conditioning was performed 24 hours after training, in the absence of the tone and footshock. The conditioning chamber and test room environments were identical to those used on the training day. Cumulative time spent freezing during the 5 minute test was similarly quantified by the software. 48 hours after training, cued fear conditioning was assessed in a novel environment with distinct visual, tactile and olfactory cues. The cued test consisted of a 3 minute acclimation period followed by a 3 minute presentation of the tone CS for 3 minutes and a 90 second exploration period. Cumulative time spent freezing before and after CS presentation was quantified by the software. The chamber was cleaned with 70% ethanol between subjects.

### Morris Water Maze

Spatial learning was assessed using standard equipment and procedures as previously described [53], [63], [83], [89]. Mice were trained to find a hidden platform in a circular pool of water (120 cm diameter) filled 45 cm deep with tap water rendered opaque by the addition of white non-toxic paint (Crayola, Easton, PA). The water temperature was maintained at 23+1°C. Training consisted of 4 trials per day over 5 days. The start position and the location of the platform (NE, SE, NW, or SW) were pseudorandomized across trials. For a given subject, the hidden platform remained in the same quadrant for all trials across all training sessions. Mice were given 60 seconds to locate the hidden platform. After reaching the hidden platform, subjects were left on the platform for 15 seconds before being removed and placed under a warming light for a 1 minute intertrial interval. A mouse that failed to find the platform within the time limit was ascribed an escape latency of 60 seconds and guided to the platform by the experimenter. Trials were videotaped and scored with WaterMaze video tracking software (Actimetrics, Inc., Wilmette, IL). Latency to find the platform, average swim speed (total cm distance traveled/seconds to reach the platform), and thigmotaxis (percent time spent in the outer 8 cm annulus at the perimeter of the pool) were automatically measured for each training trial. Hidden platform training continued until the *En2*+/+ group met the latency criterion of 15 seconds or less to find the hidden platform.

Mice were tested on a 60 second probe trial 2–3 hours after completing hidden platform testing on the day in which the latency criterion was met. Mice were placed into the pool in the quadrant opposite to the quadrant containing the platform during training. Percent time spent in each quadrant, the number of crossings over the trained platform location and the corresponding regions in non-trained quadrants, swim speed and thigmotaxis were automatically recorded. Probe trial selective search was assessed by time spent in each quadrant and the number of crossings over the trained platform location as compared to the analogous locations in the non-trained quadrants.

### Statistics

*En2*+/+, +/− and −/− littermate controls were compared for each behavioral task. Data from males and females were also compared for sex differences. When no sex differences were detected, data from males and females were combined. Genotype differences in juvenile reciprocal social interactions, elevated plus-maze, light *↔* dark exploration, male-female social interactions, self-grooming, tail suspension, forced swim and contextual fear conditioning were analyzed using one-way analyses of variance (ANOVAs), as were measures of development, general health and neurological reflexes that utilized continuous variables, such as temperature and weight. Open field locomotion, rotarod, olfactory habituation/dishabituation, acoustic startle, PPI and cued fear conditioning were analyzed with between groups repeated measures ANOVAs. Significant ANOVA results were followed by Bonferroni/Dunn post hoc tests, where applicable. Social approach results were analyzed using within groups repeated measures ANOVAs, to compare time spent in the two side chambers, and to compare time spent sniffing the novel mouse versus the novel object, within each genotype. Time spent in the center chamber is shown in the graphs for illustrative purposes only. Novel object recognition results were also analyzed using a within groups repeated measures ANOVA, to compare time spent sniffing the novel object versus the familiar object, within each genotype. Morris water maze probe trial results were similarly analyzed using a within groups repeated measures ANOVA, to compare time spent in the four quadrants within each genotype, and number of crossings over the four imaginary platform locations within each genotype. Measures of health and reflexes that utilized a rating of present or absent were analyzed for genotype differences using a Chi-squared statistic, as were data on proportion of mice reaching the learning criterion during Morris water maze hidden platform training. Reflexes or physical parameters that were rated on a 3 point ranking scale were analyzed using a non-parametric Kruskal-Wallis for ranks ANOVA. Data are presented as means ± SEMs.

## Results

### *En2* is Widely Expressed in aDult Brain Structures

**Figure 1:**
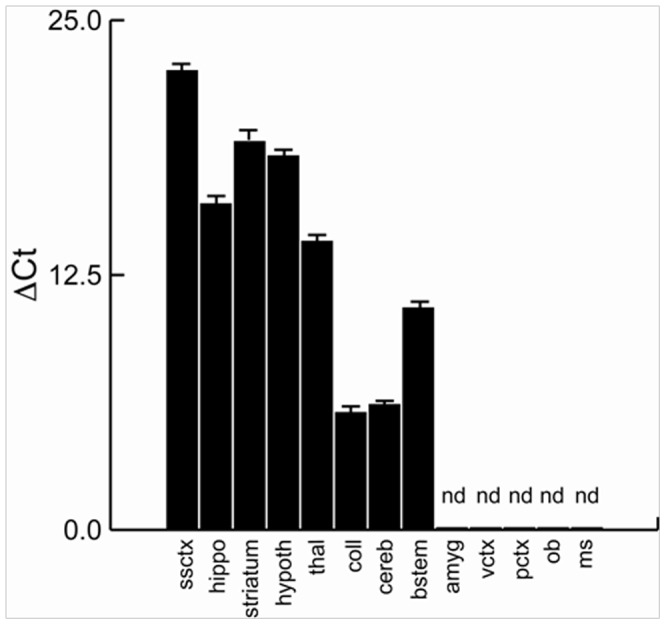
RTQPCR analysis demonstrates that *En2* is expressed in multiple adult brain structures. Average ▵Ct values with standard error are shown for the following brain structures and tissue: somatosensory cortex (ssctx), hippocampus (hippo), striatum, hypothalamus (hypoth), thalamus (thal), colliculi (coll), cerebellum (cereb), brainstem (bstem), amygdala (amyg), visual cortex (vctx), prefrontal cortex (pctx), olfactory bulb (ob), and hindlimb muscle (ms). Lower ▵Ct values indicate high gene expression, whereas higher values reflect lower levels. nd  =  none detected.

To investigate whether *En2* is expressed throughout multiple adult brain structures, we dissected the olfactory bulb, prefrontal cortex, visual cortex, somatosensory cortex, striatum, hippocampus, amygdala, hypothalamus, thalamus, colliculi, cerebellum and brainstem from *En2*+/+ adult brains and performed RTQPCR. *En2* expression was observed at the highest levels in the cerebellum, colliculi and brainstem. Lower levels of expression were detected in several forebrain structures including thalamus, hippocampus, striatum and hypothalamus. No *En2* expression was detectable in the amygdala, visual cortex, prefrontal cortex or olfactory bulb of +/+ mice (Figure 1). Since expression levels were low in some structures, we repeated the analysis in *En2*−/− mice. No signal was detected in any of the structures examined (data not shown). These results demonstrate that *En2* is widely expressed in adult brain structures.

### *En2* Heterozygous and Null Mutant Mice Display Impairments in Juvenile Social Interactions

**Figure 2:**
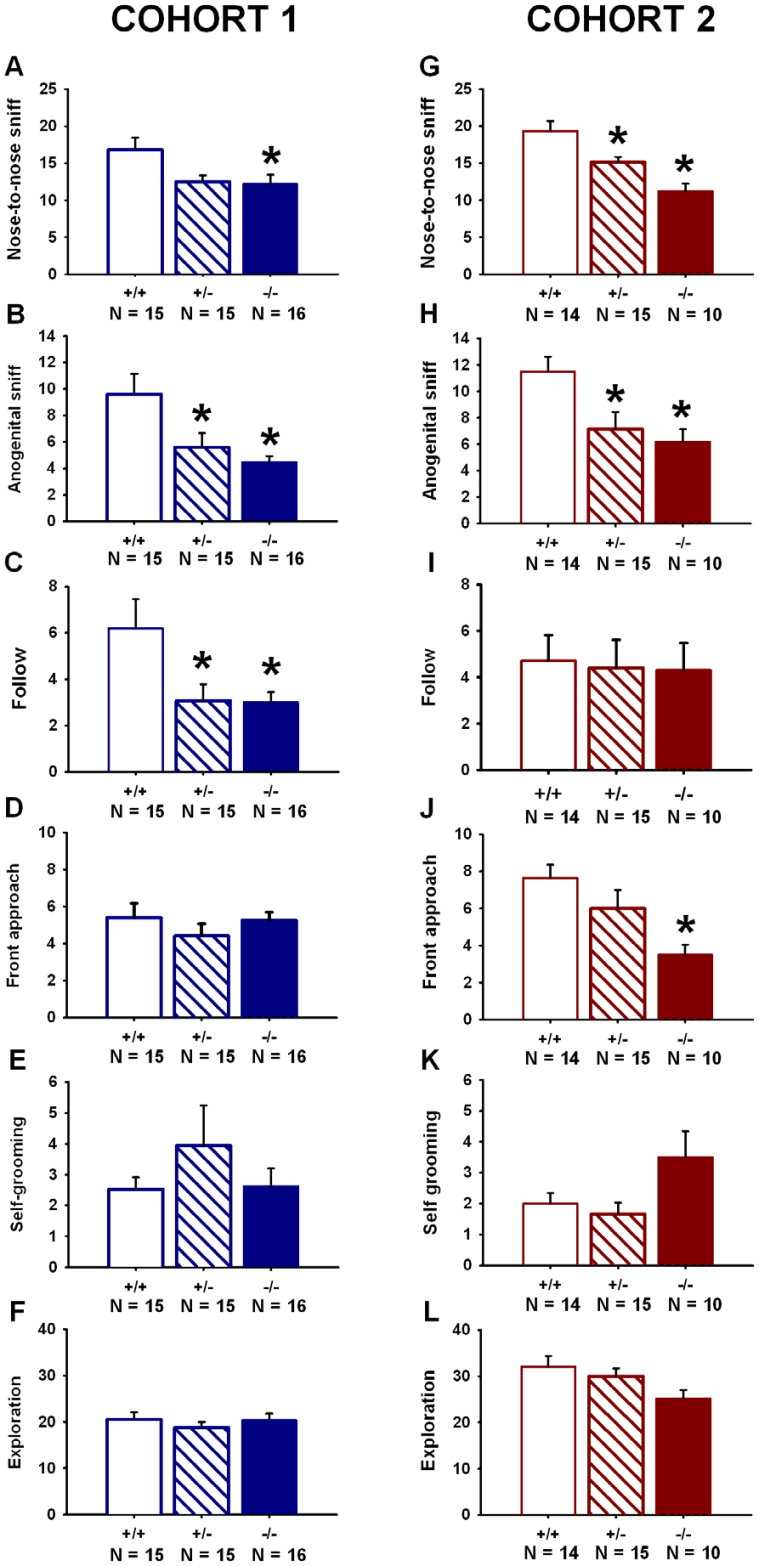
Juvenile *En2* mutant mice display fewer reciprocal social interactions, as replicated in two cohorts. Cohort 1: As compared to wildtype littermates (+/+), *En2* null mutant mice (−/−) exhibited fewer bouts of (**A**) nose-to-nose sniffing. *En2*+/− and −/− mice displayed fewer bouts of (**B**) anogenital sniffing, and (**C**) following as compared to +/+ controls. No significant genotype differences were detected in (**D**) front approach, (**E**) self-grooming, and (**F**) exploration. Cohort 2: As compared to +/+, +/− and −/− exhibited fewer bouts of (**G**) nose-to-nose sniffing and (**H**) anogenital sniffing. *En2*−/− mice exhibited fewer bouts of (**J**) front approach as compared to +/+. No significant genotype differences were detected for (**I**) following behaviors, (**K**) self-grooming, or (**L**) arena exploration. Cohort 1: N = 15+/+; N = 15+/−; N = 16−/−; Cohort 2: N = 14+/+; N = 15+/−; N = 10−/−. *p<05 vs. +/+.

Genotype differences were detected in Cohort 1 for nose-to-nose sniffing, anogenital sniffing, and following the partner mouse (Figures 2A–2C). No genotype differences were found for bouts of front approach behaviors, bouts of self-grooming behavior, or general exploration of the test arena (Figures 2D–2F). For *F* and *p* values, see Table 1. No significant genotype differences were detected for bouts of push-crawl behaviors (*F*_(2,43)_ = 1.99, *p* = 0.149; means + SEMs: 15.73+2.38 for +/+; 13.2+1.6 for +/−; 10.93+0.91 for −/−), or social grooming (*F*_(2,43)_ = 1.25, *p = *0.298; means ± SEMs: 0.47±0.22 for +/+; 0.27±0.15 for +/−; 0.75±0.27 for −/−).

Significant genotype differences were found in Cohort 2 for nose-to-nose sniffing, anogenital sniffing and front approach behaviors (Figures 2G, 2H and 2J). No genotype differences in following behaviors or bouts of exploration were found in Cohort 2 (Figures 2I and 2L). *F* and *p* values are listed in Table 1. The genotypes also did not differ on bouts of push-crawl behaviors (*F*_(2,36)_ = 0.77, *p* = 0.471; means + SEMs: 19.71±1.53 for +/+; 19.67+1.84 for +/−; 16.9±1.57 for −/−), or social grooming (*F*_(2,36)_ = 2.45, *p* = 0.101; means + SEMs: 3.86+0.88 for +/+; 2.0±0.66 for +/−; 1.8±0.53 for −/−).

**Table 1:** Statistical results of reciprocal social interactions.

Cohort	Behavioral parameters	One-way ANOVA	Post hoc test	Figure
		*F* and *p* values	*p* value	
1	Nose-to-nose sniffing	*F*(2,43) = 3.99, p = 0.026	*p = *0.015 (−/− vs. +/+)	2A
	Anogenital sniffing	*F*(2,43) = 6.13, p = 0.005	*p*<0.05 (−/− and +/− vs. +/+)	2B
	Following	*F*(2,43) = 4.50, p = 0.017	*p*<0.05 (−/− and +/− vs. +/+)	2C
	Front approach	*F*(2,43) = 0.73, p = 0.490		2D
	Self-grooming	*F*(2,43) = 0.86, p = 0.432		2E
	Exploration	*F*(2,43) = 0.51, p = 0.603		2F
2	Nose-to-nose sniffing	*F*(2,36) = 13.43, p<0.001	*p*<0.05 (−/− vs. +/+ and +/−)	2G
			*p = *0.005 (+/− vs. +/+)	
	Anogenital sniffing	*F*(2,36) = 5.58, p = 0.008	*p* <0.01 (−/− and +/− vs. +/+)	2H
	Following	*F*(2,36) = 0.033, p = 0.968		2I
	Front approach	*F*(2,36) = 5.50, p = 0.008	*p = *0.002 (−/− vs. +/+)	2J
	Self-grooming	*F*(2,36) = 3.36, p = 0.046	*p = *0.051 (−/− vs. +/+)	2K
	Exploration	*F*(2,36) = 2.46, p = 0.010		2L

Summary of statistical results of reciprocal social interactions in juvenile *En2*+/+, +/− and −/− mice paired with novel B6 partners. Data are presented in Figure 2.

### Lack of Sociability in Adult *En2* Null Mutant Mice

**Figure 3:**
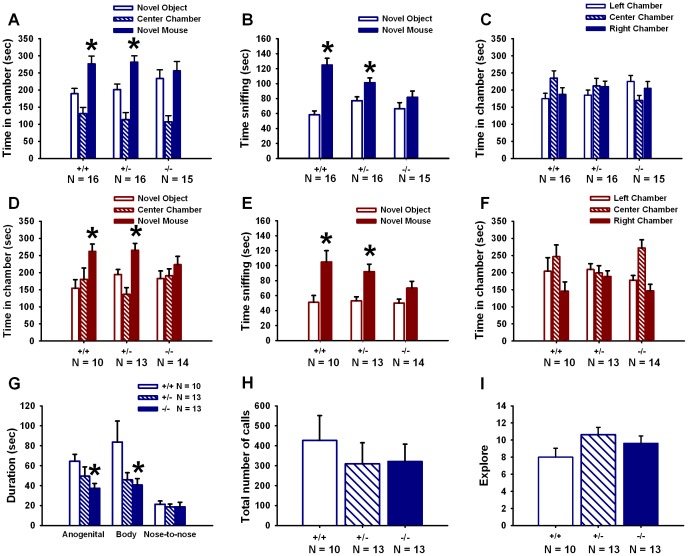
Adult *En2*−/− exhibit absence of sociability and deficits in male-female social interactions. Social approach was tested in two separate cohorts using our automated three-chambered apparatus. Cohort 1: (**A**) *En2*+/+ and +/− displayed sociability, defined as spending more time in the chamber with the novel mouse than in the chamber with the novel object. *En2*−/− did not spend more time in the novel mouse chamber as compared to the novel object chamber, meeting the definition of lack of sociability for this task. (**B**) *En2*+/+ and +/− spent more time sniffing the novel mouse than the novel object. *En2*−/− did not spend more time sniffing the novel mouse than the novel object, meeting the definition of lack of sociability on this more sensitive parameter of social interaction, and confirming results from the chamber time parameter investigation. (**C**) No genotype differences were found for time spent in each chamber during the habituation phase. Cohort 2: (**D**) Similar lack of sociability was seen in *En2*−/− mice for time spent in the novel mouse chamber vs. the novel object chamber. (**E**) *En2*−/− mice again failed to spend more time sniffing the novel mouse vs. the novel object. (**F**) Time spent in each chamber during the habituation phase was not different between genotypes. Cohort 1: N = 16+/+, N = 16+/−, N = 15−/−; Cohort 2: N = 10+/+, N = 13+/−, N = 14−/−. *p<05 vs. novel object. Reciprocal social interactions and ultrasonic vocalizations (USVs) were measured in male *En2* mice during interaction with an unfamiliar estrus female mouse. (**G**) *En2*−/− males spent less time engaged in sniffing the body and anogenital regions of the female as compared to +/+ males. (**H**) The total number of USVs emitted during the test session did not differ between genotypes. (**I**) No genotype differences were found for bouts of test cage exploration during the 5-minute test session. N = 10+/+, N = 13+/−, N = 13−/−. *p<05 vs. +/+.

In Cohorts 1 and 2, *En2*+/+ and +/− mice spent more time in the chamber containing the novel mouse than the novel object chamber (Figures 3A and 3D) and spent more time sniffing the novel mouse than the novel object (Figures 3B and 3E), indicating high sociability. *En2*−/− mice in Cohorts 1 and 2 failed to display sociability. Table 2 contains a summary of statistical results. No genotype differences were found for time spent in each chamber during the habituation phase (*p*>05 for all comparisons) (Figures 3C and 3F), indicating that there were no genotype differences in exploratory activity during this task.

**Table 2:** Statistical results of adult social approach.

Cohort	Genotype	# of animals	Chamber time	Sniff time	Sociability
			*F* and *p* values	*F* and *p* values	
1	+/+	16	*F*(1,15) = 6.47	*F*(1,15) = 34.05	Present
			*p = *0.023	*p*<0.001	
	+/−		*F*(1,15) = 8.07,	*F*(1,15) = 10.74,	Present
			*p = *0.012	*p = *0.005	
	−/−	15	*F*(1,14) = 0.20,	*F*(1,14) = 1.29,	Absent
			*p = *0.307	*p = *0.242	
2	+/+	10	*F*(1,9) = 10.71,	*F*(1,9) = 11.37,	Present
			*p = *0.010	*p = *0.008	
	+/−	13	*F*(1,12) = 5.90,	*F*(1,12) = 17.63,	Present
			*p = *0.032	*p = *0.001	
	−/−	14	*F*(1,13) = 0.93,	*F*(1,13) = 2.70,	Absent
			*p = *0.352	*p = *0.125	

Summary of statistical results of social approach behaviors in *En2*+/+, +/− and −/− mice. Data are presented in Figure 3.

### Male *En2* Null Mutants Display Reduced Social Interactions with an Estrus Female Mouse

Significant genotype differences were detected for time spent engaged in sniffing the anogenital region (*F*_(2,33)_ = 3.24, *p* = 0.05) and other body regions of the female (*F*_(2,33)_ = 3.64, *p* = 0.037) (Figure 3G). *En2*−/− spent less time engaged in anogenital and body sniffing as compared to +/+ (*p*<05 for each comparison). No genotype differences were found for time spent engaged in nose-to-nose sniffing (*F*_(2,33)_ = 0.86, *p = *0.305). All three genotypes emitted a similar number of USVs during the test session (*F*_(2,33)_ = 0.35, *p* = 0.711) (Figure 3H). Bouts of test cage exploration did not differ between genotypes (*F*_(2,33)_ = 1.24, *p = *0.302) (Figure 3I).

### *En2* Null Mutant Mice are Impaired in Contextual and Cued Fear Conditioning

**Figure 4:**
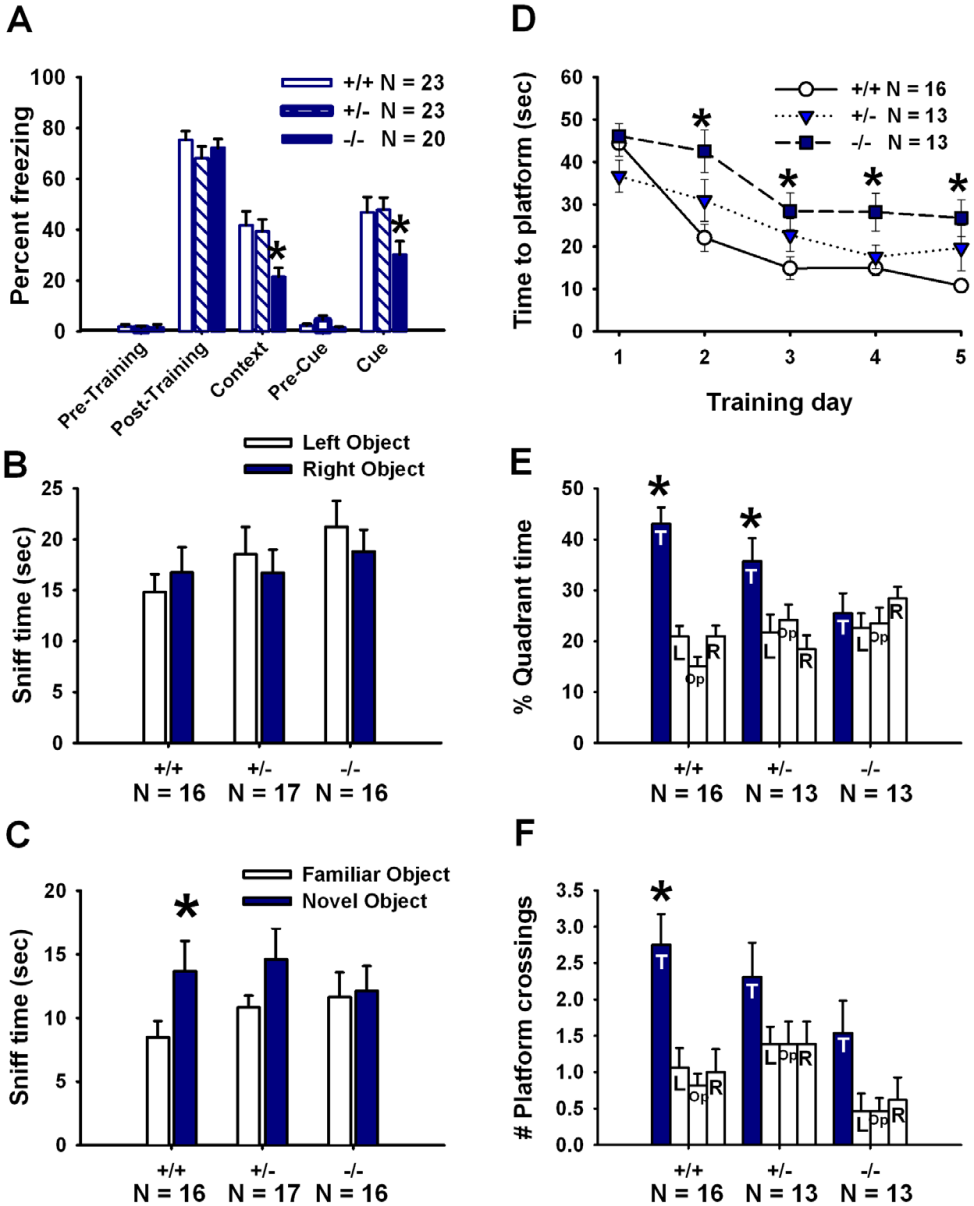
Cognitive deficits in *En2* null mutants. Cumulative time spent freezing during the fear conditioning test sessions, as quantified by the VideoFreeze software, was converted to percent time freezing for data analysis and presentation. (**A**) Despite normal postshock freezing during training, *En2*−/− exhibited significantly less freezing than *En2*+/+ and +/− mice upon testing of contextual and cued fear memory. N = 23+/+, N = 23+/−, N = 20−/−. *p<.005 vs. +/+ and +/−. (**B**) In the novel object recognition test, a lack of innate object preference was observed for *En2*+/+, +/− and −/− mice during the familiarization phase of the task. (**C**) *En2*+/+ displayed novel object recognition memory, defined as spending more time sniffing the novel object as compared to the familiar object. *En2*+/− exhibited a trend towards significant preference for the novel object, whereas −/− failed to display a preference for the novel object. N = 16+/+, N = 17+/−, N = 16−/−. *p<0.05 vs. familiar object. (**D**) In the Morris water maze, *En2*−/− showed longer latencies to reach the hidden platform during training trials as compared to +/+. *p<.01 vs. +/+. (**E**) In the probe trial, +/+ and +/− mice showed selective quadrant search with a greater percentage of time spent in the training quadrant as compared to the non-trained quadrants, while −/− failed to show selective search. (**F**) *En2*+/+ displayed a greater proportion of platform crossings in the trained quadrant as compared to the analogous locations in the non-trained quadrants, whereas +/− and −/− did not. N = 16+/+, N = 13+/−, N = 13−/−. *p<05 vs. non-trained quadrant.

All three genotypes displayed high levels of freezing subsequent to the CS–US pairings on the training day (main effect of training phase, *F*_(1, 63)_ = 653.15, *p*<001, Figure 4A). No genotype differences were detected on the training day (main effect of genotype, *F*_(2,63)_ = 0.47, *p* = 0.629; genotype × training phase interaction, *F*_(2, 63)_ = 0.21, *p* = 0.82). A genotype difference was detected for contextual fear conditioning (*F*_(2,63)_ = 5.28, *p* = 0.008). *En2*−/− displayed less freezing as compared to +/+ and +/− (*p* <0.010 for each comparison). All three genotypes displayed increased freezing following presentation of the CS on the cued day as compared to before the CS presentation (main effect of cue, *F*_(1,63)_ = 171.52, *p*<0.001). Significant genotype differences were detected for freezing in response to the cue (main effect of genotype, *F*_(2,63)_  = 6.17, *p* = 0.004; genotype × cue interaction, *F*_(1,63)_ = 5.36, *p* = 0.007). *En2*−/− mice displayed significantly less freezing upon presentation of the cue as compared to +/+ (*p* <0.005 for each comparison).

### *En2* Null Mutant Mice Fail to Display Novel Object Recognition Memory

No innate preference for object position was exhibited in *En2*+/+ (*F*_(1,15)_ = 1.51, *p* = 0.239), +/− (*F*(_1,14)_ = 1.97, *p* = 0.182), or −/− (*F*_(1,15)_ = 1.73, *p = *0.209), as indicated by similar amounts of time spent sniffing the left and right objects during the 10 minute familiarization session (Figure 4B). *En2*+/+ mice displayed a preference for the novel object over the familiar object during the 5 minute test phase (*F*_(1,15)_ = 4.88, p = 0.043) (Figure 4C). A trend towards a significant preference for the novel object over the familiar object was detected for +/− mice (*F*_(1,17)_ = 3.05, p = 0.099). *En2*−/− mice failed to display a preference for the novel object over the familiar object (*F*_(1,15)_ = 0.07, *p = *0.801). Total time spent sniffing the two objects, used as a measure of general object exploration, did not differ between genotypes (*F*_(2,46)_ = 0.28, *p = *0.757).

### *En2* Null Mutant Mice Display Spatial Learning Deficits

Latency to escape to the hidden platform decreased over the training days for all three genotypes (main effect of training day, *F*_(4,156)_ = 39.20, *p*<001). Genotype differences were detected for latency to escape to the hidden platform (main effect of genotype, *F*_(2,39)_ = 5.91, *p* = 0.006; genotype × training day interaction, *F*_(8,156)_ = 2.79, *p* = 0.014) (Figure 4D). Escape latencies for each training day differed across genotypes on training days 2 (*F*_(2,39)_ = 6.11, *p* = 0.005), 3 (*F*_(2,39)_ = 3.70, *p = *0.034), 4 (*F*_(2,39)_ = 5.63, *p* = 0.010), and 5 (*F*_(2,39)_ = 4.21, *p* = 0.025). *En2*−/− mice displayed longer latencies to escape over all of these training days as compared to +/+ controls (*p* ≤0.01 for each comparison). A greater proportion of +/+ reached the 15 second latency criterion by the fifth day of hidden platform training as compared to −/− (*Χ* ^2^_(2)_ = 8.51, *p* = 0.014). Genotypes did not differ on swim speed (main effect of genotype, *F*_(2,39)_ = 2.44, *p* = 0.101; genotype × training day interaction, *F*_(6,117)_ = 0.25, *p* = 0.958) or time spent in the perimeter of the pool (main effect of genotype, *F*_(2,39)_ = 0.63, *p* = 0.534; genotype × training day interaction, *F*_(6,117)_ = 1.44, *p* = 0.205) over hidden platform training.

On the probe trial, *En2*+/+ and +/− mice spent a greater proportion of time in the previously trained quadrant than in the three untrained quadrants (*F*_(3,45)_ = 9.24, *p*<0.001 for +/+; *F*_(3,36)_ = 3.42, *p* = 0.027 for +/−), indicating selective quadrant search. *En2*−/− mice did not spend more time in the trained quadrant as compared to the untrained quadrants (*F*_(3,36)_ = 0.52, *p* = 0.674), indicating a lack of selective quadrant search (Figure 4E). Similarly, *En2*+/+ mice made a greater number of crossings over the former location of the hidden platform as compared to analogous locations in the untrained quadrants (*F*_(3,45)_ = 9.24, *p*<0.001), again indicating selective quadrant search (Figure 4F). Number of platform crossings in the trained quadrant as compared to analogous locations in the non-trained quadrants was not significantly different for *En2*+/− (*F*_(3,36)_ = 1.82, *p* = 0.160) or −/− mice (*F*_(3,36)_ = 2.92, *p = *0.074). A genotype difference was detected for total number of platform crossings made during the probe trial (*F*_(2,39)_ = 6.28, *p* = 0.005). *En2*−/− mice made fewer total crossings as compared to +/+ and +/− (*p* ≤0.01 for each comparison). No genotype differences were detected for swim speed (*F*_(2,39)_ = 0.95, *p* = 0.394) or time spent near the perimeter of the pool during the 60-second probe trial (*F*_(2,39)_ = 1.06, *p* = 0.355).

### *En2* Null Mutant Mice Display Increased Immobility in the Forced Swim Test

**Figure 5:**
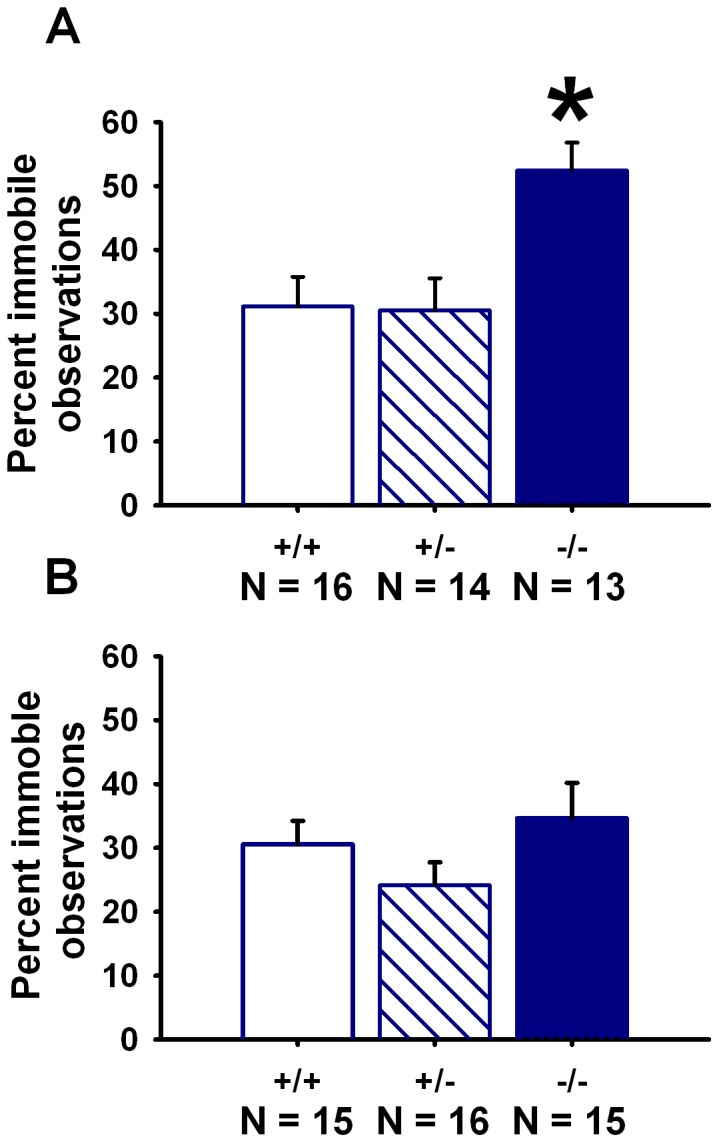
Increased depression-related behavior in *En2* null mutants. (**A**) Percentage of observations in which immobility was displayed, during the last 4 min of the forced swim test, was significantly greater for *En2*−/− as compared to +/+ and +/−. N = 16+/+; N = 14+/−; N = 13−/−. *p<.005 vs. +/+ and +/−. (**B**) No genotype differences in immobility were observed over the 6-min test session for the tail suspension test. N = 15+/+; N = 16+/−; N = 15−/−.

A genotype difference was detected in the forced swim test (*F*_(2,43)_ = 1.52, *p* = 0.004) (Figure 5A). A greater number of percent immobile observations were detected for −/− mice as compared to +/+ and +/− (*p*<0.005 for each comparison). No significant genotype differences were detected for immobility in the tail suspension test (*F*_(2,43)_ = 1.52, *p* = 0.230) (Figure 5B).

### Variable Genotype Differences in Startle Reactivity

**Figure 6:**
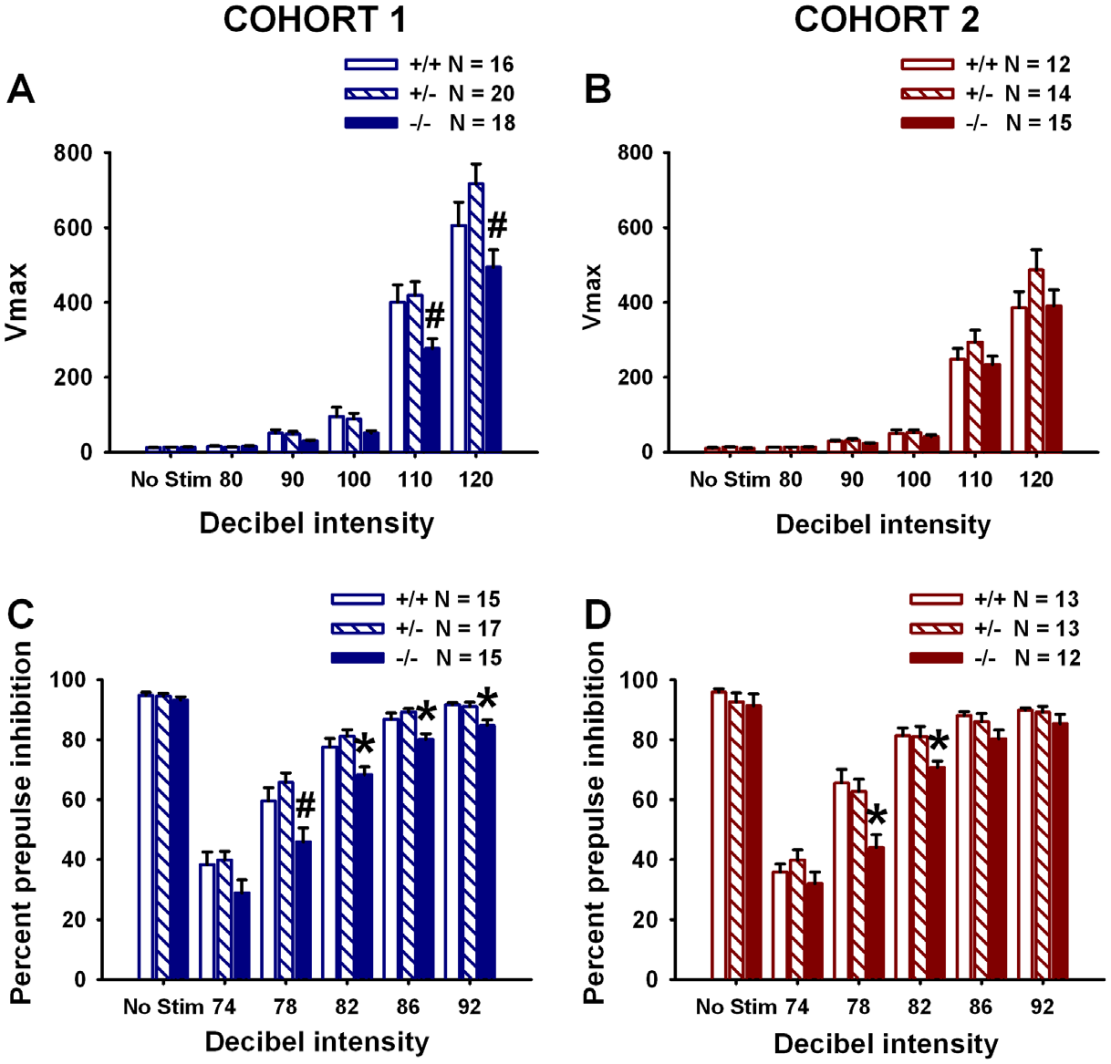
*En2* null mutants display reduced startle reactivity and reduced prepulse inhibition of acoustic startle. All three genotypes in both cohorts displayed graded startle reactivity as expected, and minimal reactivity at baseline. Cohort 1: (**A**) *En2*−/− displayed significantly lower startle responses to the 110 and 120 dB startle stimuli as compared to +/−. Cohort 2: (**B**) No genotype differences in startle reactivity were found. Cohort 1: N = 16+/+; N = 20+/−; N = 18−/−; Cohort 2: N = 12+/+, N = 14+/−, N = 15−/−. All three genotypes in both cohorts also displayed the expected increase in prepulse inhibition (PPI) of acoustic startle as a function of increasing prepulse intensity. Cohort 1: (**C**) *En2*−/− displayed significantly lower PPI as compared to +/− at the 78 dB prepulse intensity and significantly lower PPI as compared to +/+ and +/− mice at the 82, 86 and 92 dB prepulse intensities. N = 15+/+; N = 17+/−; N = 15−/−. *p<.05 vs. +/+ and +/−; #p<.05 vs. +/−. Cohort 2: (**D**) *En2*−/− displayed lower PPI as compared to +/+ at the 78 and 82 dB prepulse intensities. N = 13+/+, N = 13+/−, N = 12. *p<.05 vs. +/+.

All three genotypes displayed the expected graded startle response (main effect of stimulus intensity, Cohort 1: *F*_(5,255)_ = 299.91, *p*<0.001; Cohort 2: *F*_(5,109)_ = 196.11, *p*<0.001). Genotype differences in startle reactivity were detected in Cohort 1 (main effect of genotype, *F*_(2,51)_ = 4.89, *p* = 0.011; genotype × stimulus intensity interaction, *F*_(10,255)_ = 4.89, *p*<0.001) (Figure 6A). *F* and *p* values for each startle stimulus trial type are listed in Table 3. No genotype differences in startle reactivity were detected in Cohort 2 (main effect of genotype, *F*_(2,38)_ = 1.76, *p* = 0.186; genotype × stimulus intensity interaction, *F*_(10,190)_ = 1.24, *p* = 0.267) (Figure 6B).

**Table 3:** Statistical results for acoustic startle reactivity and prepulse inhibition of startle.

Task	Cohort	Stimulus trial	One-way ANOVA	Post hoc test	Figure
			*F* and *p* values	*p* value	
Acoustic startle	1	No stimulus	*F*(2,51) = 1.35, *p = *0.268		6A
reactivity					
		80 dB	*F*(2,51) = 0.65, *p = *0.525		
		90 dB	*F* (2,51) = 2.50, *p = *0.092		
		100 dB	*F*(2, 51) = 1.89, *p = *0.162		
		110 dB	*F*(2,51) = 4.60, *p = *0.015	*p*<0.01 (−/− vs. +/−)	
		120 dB	*F*(2,51) = 4.43, *p = *0.017	*p*<0.005 (−/− vs. +/−)	
	2	No stimulus	*F*(2,38) = 2.08, *p = *0.139		6B
		80 dB	*F*(2,38) = 0.23, *p = *0.799		
		90 dB	*F*(2,38) = 2.10, *p = *0.137		
		100 dB	*F*(2,38) = 0.66, *p = *0.521		
		110 dB	*F*(2,38) = 1.30, *p = *0.283		
		120 dB	*F*(2,38) = 1.43, *p = *0.250		
Prepulse	1	No stimulus	*F*(2,44) = 0.73, *p = *0.486		6C
inhibition					
		74 dB	*F*(2,44) = 2.35, *p = *0.107		
		78 dB	*F*(2,44) = 6.12, *p*<0.005	*p*<0.002 (−/− vs. +/−)	
		82 dB	*F*(2,44) = 6.80, *p*<0.003	*p = *0.015 (−/− vs. +/+)	
				*p*<0.001 (−/− vs. +/−)	
		86 dB	*F*(2,44) = 7.61, *p*<0.002	*p = *0.009 (−/− vs. +/+)	
				*p*<0.001 (−/− vs. +/−)	
		92 dB	*F*(2,44) = 7.14, *p = *0.002	*p*<0.002 (−/− vs. +/+)	
				*p*<0.003 (−/− vs. +/−)	
	2	No stimulus	*F*(2,35) = 0.66, *p = *0.532		6D
		74 dB	*F*(2,35) = 1.42, *p = *0.255		
		78 dB	*F*(2,35) = 6.94, *p*<0.003	*p*<0.002 (−/− vs. +/+)	
				*p = *0.005 (−/− vs. +/−)	
		82 dB	*F*(2,35) = 4.50, *p = *0.018	*p = *0.012 (−/− vs. +/+)	
				*p = *0.014 (−/− vs. +/−)	
		86 dB	*F*(2,35) = 2.69, *p = *0.082		
		92 dB	*F*(2,35) = 1.21, *p = *0.311		

Summary of statistical results of acoustic startle reactivity and prepulse inhibition of startle in *En2*+/+, +/− and −/− mice. Data are presented in Figure 6.

### *En2* Null Mutant Mice Display Reduced Prepulse Inhibition of Acoustic Startle

All three genotypes displayed increased inhibition of startle with increasing prepulse intensity (main effect of prepulse intensity, Cohort 1: *F*_(5,220)_ = 297.63, *p*<0.001; Cohort 2: *F*_(5,175)_ = 237.28, *p*<0.001). Genotype differences in PPI were also detected (main effect of genotype, Cohort 1: *F*_(2,44)_ = 8.83, *p*<0.001; Cohort 2: *F*_(2,35)_ = 5.27, *p* = 0.01; genotype × prepulse intensity interaction, Cohort 1: *F*_(10,220)_ = 2.04, *p* = 0.031; Cohort 2: *F*_(10, 175)_ = 2.41, *p* = 0.012) (Figures 6C and 6D). Genotype differences in PPI were detected for selected prepulse trials. Table 3 lists *F* and *p* values for specific trials.

### *En2* Null Mutant Mice Display Mild Impairments in Motor Abilities

**Figure 7:**
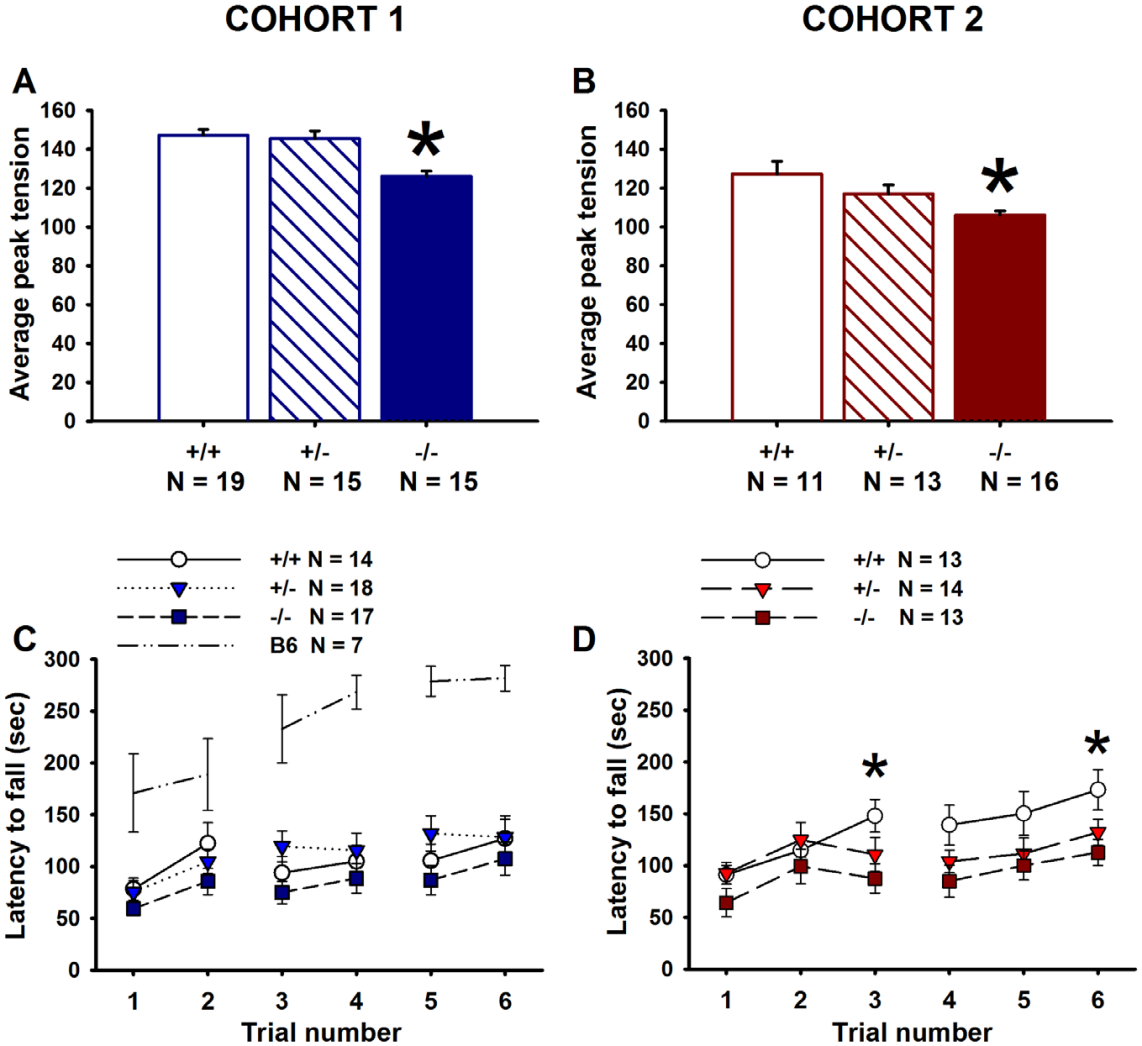
*En2* mice display deficits in forelimb grip strength and in rotarod motor learning and coordination under certain testing conditions. Cohort 1: (**A**) Grip strength was reduced in *En2*−/− as compared to +/+ and +/−. N = 19+/+, N = 15+/−, N = 15−/−; *p<.005 vs. +/+ and +/−. Cohort 2: (**B**) *En2*−/− displayed reduced grip strength as compared to +/+ only. N = 11+/+, N = 13+/−, N = 16−/−. *p<005 vs. +/+. Mice were tested for rotarod motor coordination and learning over a total of 6 trials. Cohort 1: (**C**) Mice were given two trials per day for three days, with a 60 minute intertrial interval. No genotype differences were observed in latency to fall from the rotarod. Mean latency to fall for the standard C57BL6/J (B6) strain is shown as an illustrative comparison. Cohort 2: (**D**) Mice were given three trials per day for two days, with a 30 minute intertrial interval. *En2*−/− displayed lower latencies to fall as compared to +/+ on trials 3 and 6. Cohort 1: N = 14+/+, N = 18+/−, N = 17−/−, N = 7 C57BL6/J; Cohort 2: N = 13+/+, N = 14+/−, N = 13−/−. *p<.05 vs. +/+.

Grip strength differed across genotypes in both cohorts (main effect of genotype, Cohort 1: *F*_(2,43)_ = 12.08, *p*<0.001; Cohort 2: F_(2,34)_ = 10.02, *p*<0.001). Grip strength was reduced in *En2*−/− mice as compared to both +/+ and +/− mice for Cohort 1 (*p*<0.001 for each comparison) (Figure 7A). *En2*−/− mice displayed reduced grip strength as compared to +/+ mice only for Cohort 2 (*p* = 0.001) (Figure 7B), though a trend toward was observed in comparison to +/− (*p* = 0.068). Males of both cohorts exhibited greater grip strength as compared to females (main effect of sex, Cohort 1: *F*_(1,43)_ = 12.40, *p*<001; Cohort 2: *F*_(1,34)_ = 11.95, *p* = 0.002).

Figures 7C and 7D illustrate performance on the accelerating rotarod test of motor coordination and balance in two cohorts of adult *En2* mice, which were tested under slightly different conditions. Rotarod data from C57BL6/J mice are shown for comparative purposes, as these mice were not littermates of the *En2* mice. As expected, latency to fall increased over the six trials independent of genotype in Cohorts 1 and 2 (main effect of trial, Cohort 1: *F*_(5,230)_ = 9.82, *p*<000; Cohort 2: *F*_(5,185)_ = 8.79, *p*<0001). No genotype differences were found for latency to fall in Cohort 1 (main effect of genotype, *F*_(2,37)_ = 1.53, *p* = 0.227) (Figure 7C). A genotype difference was detected in Cohort 2 (main effect of genotype, *F*_(2,27)_ = 3.72, *p* = 0.034) (Figure 7D). Genotype differences were detected for latency to fall on trials 3 (*F*_(2,37)_ = 3.74, *p* = 0.033) and 6 (*F*_(2,37)_  = 4.08, *p = *0.025). *En2*−/− mice displayed lower latencies to fall as compared to +/+ mice during these trials (*p* <0.01 for each comparison). Near-significant trends toward lower latencies to fall were detected for *En2*+/− mice as compared to +/+ on these trials (*p*<0.10 for each comparison). A trend toward a genotype difference was found for trial 4 (*F*_(2,37)_  = 3.13, *p = *0.055).

### *En2* Mutant Mice do not Display an Anxiety-like Phenotype

**Figure 8:**
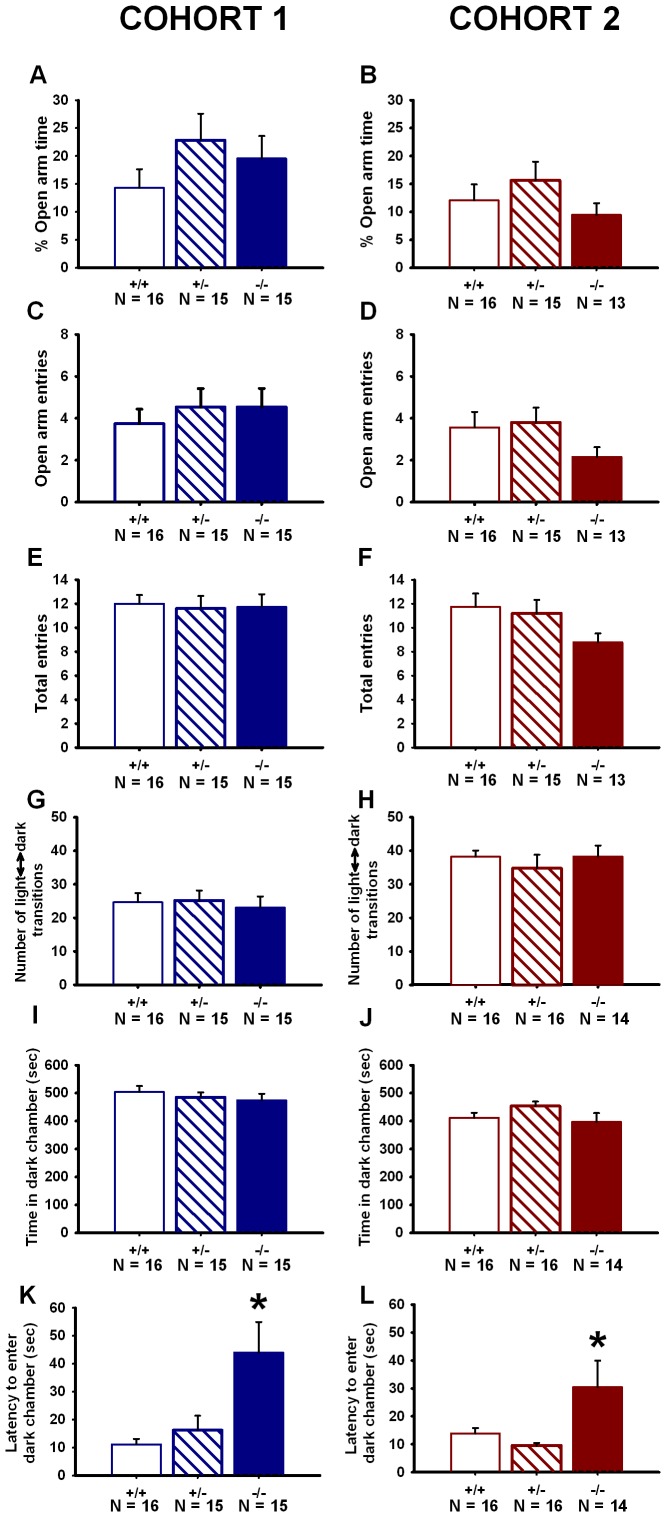
Anxiety-like behaviors are normal in *En2* null mutants. On the elevated plus-maze, no genotype differences were seen in (**A**–**B**) percent open arm time, (**C**–**D**) number of open arm entries, or (**E**–**F**) total number of arm entries. Cohort 1: N = 16+/+; N = 15+/−; N = 15−/−; Cohort 2: N = 16+/+, N = 15+/−, N = 13−/−. In the light ↔ dark exploration task, no genotype differences were observed in (**G**–**H**) the number of transitions between the light and dark chambers or (**I**–**J**) time spent in the dark chamber. In Cohort 1 (**K**) and Cohort 2 (**L**), *En2*−/− mice displayed a higher mean latency to enter the dark chamber as compared to +/− and −/− mice. Latency to enter the dark chamber is not a standard parameter for anxiety-like traits or responses to anxiolytic drugs in this task, but may instead reflect the somewhat lower exploratory activity in −/−, as shown in Figure 8. Cohort 1: N = 16+/+; N = 15+/−; N = 15−/−; Cohort 2: N = 16+/+, N = 16+/−, N = 14−/−. *p<.005 vs. +/+ and +/−.

No significant genotype differences were detected for percentage of time spent on the open arms of the plus-maze (Figures 8A and 8B), entries into the open arm (Figures 8C and 8D), or total entries into the open and closed arms (Figures 8E and 8F). For the light ↔ dark exploration test, no significant genotype differences were detected for number of light ↔ dark transitions (Figures 8G and 8H) or time spent in the dark chamber (Figures 8I and 8J). A genotype difference was found for latency to enter the dark chamber from the light chamber (Figures 8K and 8L). *En2*−/− mice displayed a longer latency to enter the dark chamber as compared to +/+ and +/− mice in Cohort 1 and as compared to +/− mice only in Cohort 2. Latency to enter the dark chamber is thought to reflect exploratory activity, and is not the standard parameter for anxiety-like traits or responses to anxiolytic drugs in this task. *F* and *p* values for all genotype comparisons are listed in Table 4.

**Table 4:** Statistical results for anxiety-like behaviors.

Cohort	Task	Behavioral parameter	One-way ANOVA	Post hoc test	Figure
			*F* and *p* values	*p* value	
1					
	Elevated	Time spent in open arm	*F*(2,43) = 1.15,		8A
	plus-maze	(% of total time)	*p = *0.327		
		Entries into open arm	*F*(2,43) = 0.32,		8C
			*p = *0.731		
		Total open and closed arm	*F*(2,43) = 0.05,		8E
		entries	*p = *0.955		
	Light ↔ dark	Number of light ↔ dark	*F*(2,43) = 0.13,		8G
	exploration	transitions	*p = *0.088		
		Time spent in the dark	*F*(2,43) = 0.53,		8I
		chamber	*p = *0.590		
		Latency to enter the dark	*F*(2,43) = 6.56,	*p = *0.030 (−/− vs. +/+)	8K
		chamber	*p = *0.003	*p = *0.007 (−/− vs. +/−)	
2					
	Elevated	Time spent in open arm	*F*(2,41) = 1.11,		8B
	plus-maze	(% of total time)	*p = *0.338		
		Entries into open arm	F(2,41) = 2.23,		8D
			*p = *0.120		
		Total open and closed arm	*F*(2,41) = 1.65,		8F
		entries	*p = *0.205		
	Light ↔ dark	Number of light ↔ dark	*F*(2,43) = 0.41,		8H
	exploration	transitions	*p = *0.667		
		Time spent in the dark	*F*(2,43) = 0.53,		8J
		chamber	*p = *0.590		
		Latency to enter the dark	F(2,43) = 4.38,	*p*<0.005 (−/− vs. +/+)	8L
		chamber	*p = *0.019	*p = *0.007 (−/− vs. +/−)	

Summary of statistical results of elevated-plus maze and light ↔ dark exploration. Data are presented in Figure 8.

### Exploratory Activity is Normal in *En2* Mutant Mice

**Figure 9:**
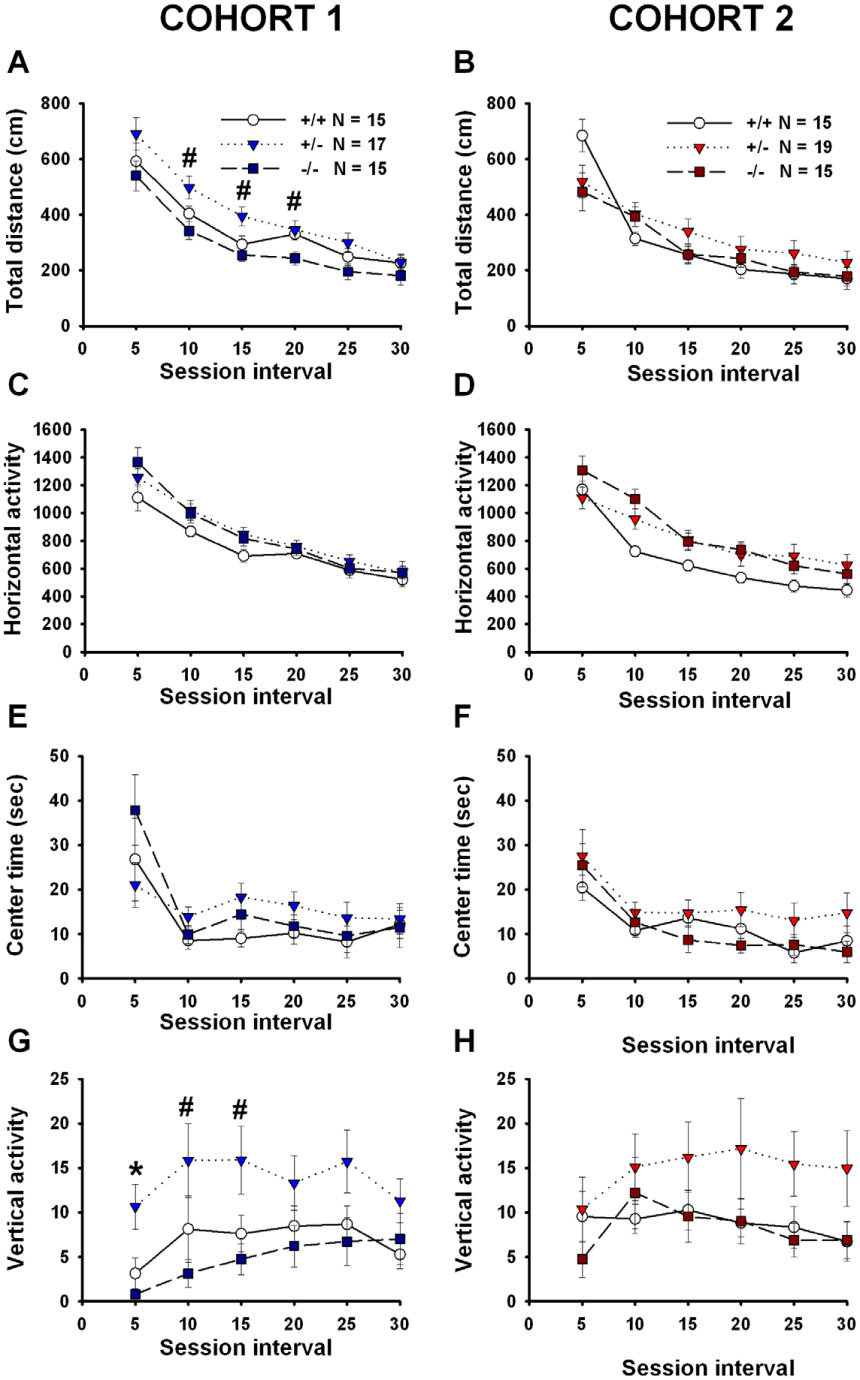
Genotype differences in selected parameters of open field activity in adult *En2* mice. Cohort 1: *En2*−/− mice (**A**) traversed less total distance as compared to +/− and (**G**) exhibited fewer bouts of vertical activity as compared to +/+ and +/− mice. No genotype differences were detected for (**C**) horizontal activity or (**E**) center time. N = 15+/+; N = 17+/−; N = 15−/−. *p<.05 vs. +/+ and +/−; #p<.005 vs. +/−. Cohort 2: *En2*+/− and −/− mice exhibited greater (**D**) horizontal activity as compared to +/+ mice during minutes 6–10 of the test session. No genotype differences were found for (**B**) total distance traveled, (**F**) center time or (**H**) vertical activity. N = 15+/+, N = 19+/−, N = 15−/−. *p<05 vs. +/+.

All three genotypes displayed decreases in total distance traveled (main effect of time, Cohort 1: *F*_(5,220)_ = 68.31, *p*<0.001; Cohort 2: *F*_(5,230)_ = 85.66, *p*<0.001) (Figures 9A and 9B), horizontal activity (Cohort 1: *F*_(5,220)_ = 83.46, *p*<0.001; Cohort 2: *F*_(5,230)_ = 137.18, *p*<0.001) (Figures 9C and 9D), center time (Cohort 1: *F*_(5,220)_ = 12.52, *p = *0.050; Cohort 2: *F*_(5, 230)_ = 14.32, *p*<0.001) (Figures 9E and 9F) and vertical activity (Cohort 1: *F*_(5,220)_ = 4.12, *p* = 0.001; Cohort 2: *F*_(5,230)_ = 2.16, *p* = 0.050) (Figures 9G and 9H) over the 30 minute test session, reflecting normal habituation to the novel open field. In Cohort 1, genotype differences were detected for total distance traveled and vertical activity. In Cohort 2, genotype differences were detected for horizontal activity. *F* and *p* values for genotype comparisons of total distance traveled, horizontal activity, and vertical activity are listed in Table 5.

**Table 5:** Statistical results for selected parameters of open field locomotor activity.

Parameter	Session	Cohort 1	Cohort 1	Cohort 2	Cohort 2	Figures
	Interval	One-way ANOVA	Post hoc test	One-way ANOVA	Post hoc test	
	(min)	*F* and *p* value	*p* value	*F* and *p* value	*p* value	
Total distance	1–5	F(2,44) = 1.75,		F(2,46) = 2.88,		9A–B
traveled		*p = *0.186		*p = *0.067		
	6–10	*F*(2,44) = 5.20,	*p = *0.010	*F*(2,46) = 1.64,		
		*p = *0.009	(−/− vs. +/−)	*p = *0.205		
	11–15	*F*(2,44) = 5.94,	*p*<0.002	*F*(2,46) = 1.68,		
		*p = *0.005	(−/− vs. +/−)	*p = *0.199		
	16–20	*F*(2,44) = 4.09,	p = 0.010	*F*(2,46) = 0.91,		
		*p = *0.023	(−/− vs. +/−)	*p = *0.411		
	21–25	*F*(2,44) = 2.12,		*F*(2,46) = 1.20,		
		*p = *0.078		*p = *0.311		
	26–30	*F*(2,44) = 0.83,		*F*(2,46) = 0.66,		
		*p = *0.441		*p = *0.520		
Horizontal	1–5	*F*(2,44) = 2.00,		*F*(2,46) = 1.52,		9C–D
activity						
		*p = *0.148		*p = *0.229		
	6–10	*F*(2,44) = 1.72,		*F*(2,46) = 7.70,	*p*<0.001	
		*p = *0.190		*p = *0.001	(−/− vs. +/+)	
					*p*<0.015	
					(−/− vs. +/−)	
	11–15	*F*(2,44) = 2.82,		*F*(2,46) = 2.82,		
		*p = *0.071		*p = *0.070		
	16–20	*F*(2,44) = 0.37,		*F*(2,46) = 2.62,		
		*p = *0.691		*p = *0.083		
	21–25	*F*(2,44) = 0.49,		*F*(2,46) = 2.85,		
		*p = *0.618		*p = *0.068		
	26–30	*F*(2,44) = 0.25,		*F*(2,46) = 1.57,		
		*p = *0.780		*p = *0.219		
Vertical activity	1–5	*F*(2,44) = 7.84,	*p*<0.006	*F*(2,46) = 0.97,		9G–H
		*p = *0.001	(−/− vs. +/+)	*p = *0.388		
			p<0.001			
			(−/− vs. +/−)			
	6–10	*F*(2,44) = 3.56,	*p*<0.002	*F*(2,46) = 0.75,		
		*p = *0.037	(−/− vs. +/−)	*p = *0.478		
	11–15	*F*(2,44) = 4.39,	*p*<0.007	*F*(2,46) = 1.32,		
		*p = *0.018	(−/− vs. +/−)	*p = *0.278		
	16–20	*F*(2,44) = 1.94,		*F*(2,46) = 1.43,		
		*p = *0.155		*p = *0.250		
	21–25	*F*(2,44) = 2.72,		*F*(2,46) = 2.63,		
		*p = *0.077		*p = *0.830		
	26–30	*F*(2,44) = 1.75,		*F*(2,46) = 2.19,		
		*p = *0.186		*p = *0.123		

Summary of statistical results of selected parameters of open field locomotor activity. Data are presented in Figure 9.

### Normal Olfactory Abilities in *En2* Mutant Mice

**Figure 10:**
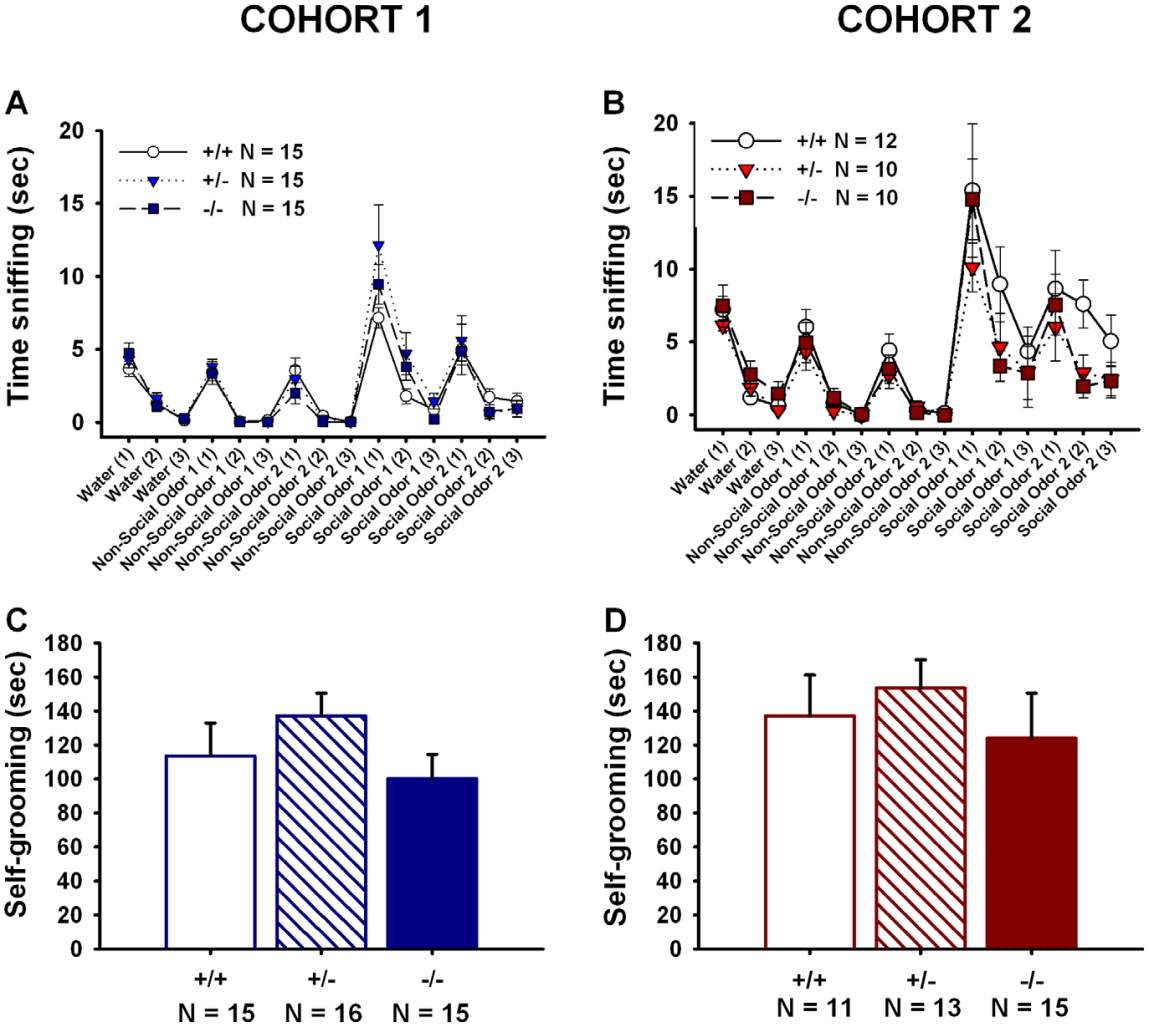
No genotype differences in olfactory habituation/dishabituation to social and non-social odors or repetitive self-grooming. In cohort 1 (**A**) and cohort 2 (**B**), a significant decline in sniffing (habituation) to repeated presentations of water, two non-social odors and two social odors was observed all three genotypes. A significant increase in sniffing upon the first presentation of a novel odor (dishabituation) was also observed across genotypes. Cohort 1: N = 15+/+, N = 15+/−, N = 15−/−; Cohort 2: N = 12+/+, N = 10+/−, N = 10−/−. *En2*+/+, +/− and −/− mice in Cohort 1 (**C**) and Cohort 2 (**D**) spent a similar amount of cumulative time engaged in self-grooming during a 10 min test session. Cohort 1: N = 15+/+, N = 16+/−, N = 15−/−; Cohort 2: N = 11+/+, N = 13+/−, N = 15−/−.

All three genotypes in Cohorts 1 (Figure 10A) and 2 (Figure 10B) displayed olfactory habituation as indicated by the decline in time spent sniffing on repeated exposures to water, non-social odor 1, non-social odor 2, social odor 1, and social odor 2. No genotype differences were detected across the trials. All three genotypes displayed dishabituation upon presentation of a new odor as indicated by increases in time spent sniffing from presentation of water to non-social odor 1, non-social odor 1 to non-social odor 2, non-social odor 2 to social odor 1, and social odor 1 to social odor 2. No genotype differences in dishabituation to a new odor were detected. *F* and *p* values for specific habituation and dishabituation trials are listed in Table 6.

**Table 6:** Statistical results for olfactory habituation/dishabituation.

Cohort	Genotype	Habituation to water	Dishabituation water to non-social odor 1	Habituation to social odor 1	Dishabituation non-social odor 1 to non-social odor 2	Habituation to non-social odor 2	Dishabituation to non-social odor 2 social odor 1	Habituation to social odor 1	Dishabituation social odor 1 tosocial odor 2	Habituation to social odor 2
1	+/+	*p*<0.001	*p*<0.001	*p*<0.001	*p*<0.005	*p*<0.001	*p*<0.001	*p*<0.001	*p*<.05	*p*<.05
	+/−	*p*<0.001	*p*<0.001	*p*<0.001	*p*<0.01	*p*<0.001	*p*<0.001	*p*<0.001	*p*<.05	*p*<0.005
	−/−	*p*<0.001	*p*<0.001	*p*<0.001	p<0.05	*p*<0.005	*p*<0.001	*p*<0.001	*p*<0.001	*p*<0.001
	Genotype	NS	NS	NS	NS	NS	NS	NS	NS	NS
	difference									
2	+/+	*p*<0.001	*p*<0.001	*p*<0.001	*p*<0.005	*p*<0.001	*p*<0.01	*p*<0.05	*p*<0.05	*p*<0.05
	+/−	*p*<0.001	*p*<0.01	*p*<0.005	*p*<0.05	*p*<0.005	*p*<0.001	*p*<0.05	*p*<0.05	*p*<0.05
	−/−	*p*<0.005	*p*<0.01	*p*<0.005	*p*<0.01	*p*<0.005	*p*<0.001	*p*<0.001	*p*<0.05	*p*<0.05
	Genotype	NS	NS	NS	NS	NS	NS	NS	NS	NS
	difference									

Summary of statistical results of the olfactory habituation/dishabituation test. Data are presented in Figure 10.

### No Genotype Differences in Repetitive Self-grooming Behavior

Figures 10C and 10D illustrate time spent engaged in repetitive self-grooming by two separate cohorts of adult *En2* mice. No genotype differences were detected for time spent self-grooming in either cohort (Cohort 1: *F*_(2,43)_ = 1.43, *p* = 0.250; Cohort 2: *F*_(2,33)_ = 1.13, *p* = 0.334).

### Normal Neurobehavioral Development in *En2* Mutant Mice

**Figure 11:**
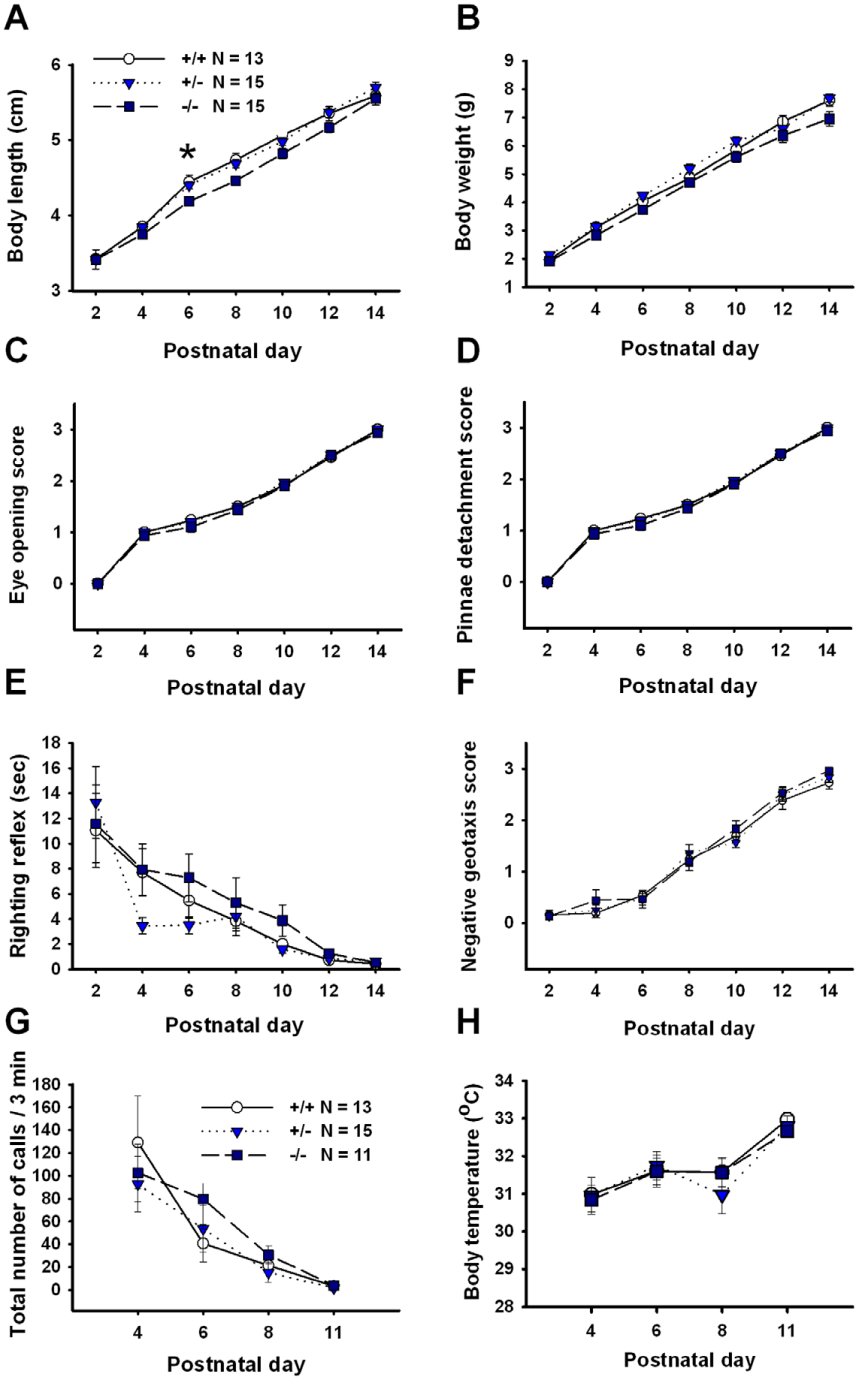
Developmental milestones and pup ultrasonic vocalizations are generally normal in *En2* null mutants. Developmental milestones were measured every other day from postnatal day 2 to 14, using a modified version of the Fox battery. *En2*−/− pups exhibited reduced (**A**) body length as compared to +/+ pups on postnatal day 6 only. No genotype differences were observed in (**B**) body weight, (**C**) eye opening, (**D**) pinnae detachment, (**E**) righting reflex, and (**F**) negative geotaxis. N = 13+/+, N = 15+/−, N = 15. *p<05 vs. +/+. Pup ultrasonic vocalizations to separation from the mother and siblings were measured over postnatal days 4, 6, 8 and 11. (**G**) Number of calls emitted during the separation period did not differ across genotypes on any the four days of testing. (**H**) Body temperature did not differ between genotypes. N = 13+/+, N = 15+/−, N = 11−/−.

All three genotypes displayed proper growth and reflex development as indicated by significant main effects of postnatal day for body length (*F*_(6,240)_ = 575.61, *p*<0.001) (Figure 11A), body weight (*F*_(6,240)_ = 3.22, *p*<0.001) (Figure 11B), eye opening (*F*_(6,240)_ = 232.69, *p*<0.001) (Figure. 11C), pinnae detachment (*F*_(6,240)_ = 2163.69, *p*<0.001) (Figure 11D), righting reflex (*F*_(6,240)_ = 660.35, *p*<0.001) (Figure 11E) and negative geotaxis (*F*_(6,240)_ = 264.79, *p*<0.001) (Figure 11F). A genotype difference was found for body length (main effect of genotype, *F*_(2,40)_ = 3.70, *p* = 0.034). *En2*−/− displayed reduced body length as compared to +/+ littermates on pnd 6 only (*p* = 0.010). A trend toward a significant genotype difference was detected for body weight (*F*_(2,40)_ = 3.22, *p* = 0.051). No genotype differences were detected for eye opening (*F*_(2,40)_ = 0.48, *p* = 0.624), pinnae detachment (*F*_(2,40)_ = 0.85, *p* = 0.433), righting reflex (*F*_(2,40)_ = 1.57, *p* = 0.220) or negative geotaxis (*F*_(2,40)_ = 0.45, *p* = 0.640).

### No Genotype Differences in Pup Ultrasonic Vocalizations

The number of calls emitted during the 3 minute test session decreased over postnatal days 4–11 independent of genotype (main effect of day, *F*_(3,108)_ = 16.34, *p*<0.001). No genotype differences were detected for mean total number of calls (main effect of genotype, *F*_(2,36)_ = 0.56, *p* = 0.577) (Figure 11G). All three genotypes displayed the expected increases in body weight over postnatal days 4–11 (main effect of day, *F*_(3,108)_ = 1079.02, *p*<0.00) and body temperature (*F*_(3,108)_ = 16.93, *p*<.0001) (Figure 11H).

### Normal General Health and Pain Sensitivity in *En2* Mutant Mice

General health and sensitivity to painful stimuli were assessed in two separate cohorts of *En2* adult mice. Table 7 lists scores for measures of general health and pain sensitivity for Cohort 1 only. No genotype differences were detected for body weight (*F*_(2,42)_ = 0.94, *p* = 0.398 for Cohort 1; *F*_(2,39)_ = 1.19, *p* = 0.317 for Cohort 2), or body temperature (*F*_(2,42)_ = 1.42, *p* = 0.252 for Cohort 1; *F*_(2,39)_ = 0.82, *p* = 0.449 for Cohort 2). Appearance of the fur, body tone, limb tone and skin color were also similar across genotypes (*p*>0.792 for each comparison in Cohorts 1 and 2). No obvious physical abnormalities were seen in any of the mice. A significant sex difference was detected for body weight, with males displaying higher body weights than females independent of genotype (significant main effect of sex, *F*_(1,42)_ = 50.34, *p*<0.001 for Cohort 1; (*F*_(1,46)_ = 29.26, *p*<0.001 for Cohort 2). All 3 genotypes displayed normal reflexes including eye blink, ear twitch, whisker twitch, righting reflex, Preyer startle response as a measure of hearing, and forepaw reaching (*Χ* ^2^_(2)_<5.51, *p*>0.064 for all comparisons in Cohorts 1 and 2). No genotype differences were found in latency to fall in the wire hang test (Cohort 1: *F*_(2,42)_ = 0.92, *p* = 0.405; Cohort 2: *F*_(2,39)_ = 0.40, *p* = 0.673).

**Table 7:** General health, neurological reflexes, and pain sensitivity.

Genotype	+/+	+/−	−/−	Sig. Level
	N = 17	N = 16	N = 15	
**Physical Characteristics**				
Fur condition (3 pt scale)	3	3	3	NS
Bald patches (%)	5.9	6.3	6.7	NS
Missing whiskers (%)	17.7	6.3	40	NS
Piloerection (%)	0	0	0	NS
Body tone (3 pt scale)	2.8+0.10	2.9+0.09	2.7+0.12	NS
Limb tone (3 pt scale)	3	3	3	NS
Skin color (3 pt scale)	3	3	3	NS
Physical abnormalities (%)	0	0	0	NS
Body weight, males (g)	26.5+1.0	26.8+0.76	26.2+0.93	NS
Body weight, females (g)	20.4+0.63	20.9+0.62	18.6+0.79	NS
Body Temperature (°C)	36.1+0.23	35.2+0.22	35.5+0.26	NS
**Empty cage behavior**				
Transfer freezing (%)	0	0	0	NS
Wild running (%)	0	0	0	NS
Stereotypy (3 pt scale)	0	0	0	NS
Self-Grooming (3 pt scale)	0.71+0.17	1.00+0.20	1.20+0.22	NS
Exploration (3 pt scale)	2.7+0.17	2.6+0.16	2.9+0.10	NS
**Motoric abilities**				
Trunk curl (3 pt scale)	2.0+0.15	2.3+0.16	2.3+0.11	NS
Wire hang (latency sec)	59.9+0.12	59.9+0.13	58.5+1.53	NS
**Reflexes**				
Forepaw reach (%)	100	100	100	NS
Righting reflex (%)	100	100	100	NS
Corneal (%)	100	100	100	NS
Ear twitch (%)	100	100	100	NS
Whisker twitch (%)	100	100	90	NS
**Reactivity**				
Auditory Startle (%)	100	100	100	NS
Struggle/Vocalizations (%)	41.2	25.0	53.3	NS
Dowel Biting (3 pt scale)	0.65+0.17	0.38+0.16	0.33+0.16	NS
**Pain Sensitivity**				
Hot plate (latency sec)	5.4+0.57	5.4+0.42	6.2+0.48	NS
Tail flick (latency sec)	4.2+0.53	2.9+0.30	3.5+0.54	NS

Normal general health and pain responses in Cohort 1 of *En2* mice. No genotype differences were detected using a standard battery of parameters. Data shown are means ± standard error of the mean (SEM) for body weight, temperature, wire hang latency, hot plate and tail flick latency, and behaviors assessed using a 3 point ranking scale. Percentage of mice that exhibited a specific neurological reflex or physical abnormality is expressed as percent of total mice within each genotype. N = 17+/+, N = 16+/−, N = 15−/−. Similar results were obtained for Cohort 2 (data not shown).

Reactivity as measured by struggling or vocalizations and dowel biting did not differ across genotypes (*p*>0.142 for each comparison for Cohorts 1 and 2). Observations of empty cage behaviors did not reveal any genotype differences in exploration of the cage, bouts of self-grooming and stereotypy, or behaviors such as freezing or wild running upon transfer to the cage (*p*>0.196 for each comparison in Cohorts 1 and 2). No significant genotype differences were found in the latency to respond in the hot plate test (Cohort 1: *F*_(2,44)_ = 0.12, *p* = 0.493; Cohort 2: *F*_(2, 36)_ = 1.83, *p* = 0.174) or in the tail flick test (Cohort 1: *F*_(3,43)_ = 1.60, p = 0.213; Cohort 2: *F*_(2,37)_ = 1.41, *p* = 0.258 for Cohort 2) of pain sensitivity.

### Behavioral Phenotypes in *En2* Mutant Mice do not Depend on Sex

No differences were detected between males and females of *En2*+/+, +/− and −/− mice for juvenile reciprocal social interactions, adult social approach, fear conditioning, Morris water maze, forced swim, tail suspension, acoustic startle, prepulse inhibition, pup ultrasonic vocalizations, anxiety-like behaviors, open field locomotor activity, rotarod performance, olfactory habituation/dishabituation, self-grooming, pup developmental milestones and the majority of general health parameters. Sex differences were detected for two parameters, forelimb grip strength and body weight. Males displayed greater grip strength as compared to females in Cohorts 1 and 2, independent of genotype. Males also displayed higher body weights than females, with no differences between genotypes within each sex. No genotype by sex interactions were detected for any of the tasks.

## Discussion

Deletion of *En2* in mice disrupts patterning of the mid/hindbrain and produces multiple neuroanatomical and neurochemical abnormalities. Given the critical role of *En2* expression in early brain development, we sought to further understand the consequences of *En2* mutations on mouse behaviors. We investigated a comprehensive range of behavioral phenotypes in mice with heterozygous and homozygous mutations deletions in *En2*, as compared to their wildtype littermates. Cognitive deficits on three tasks, a sensorimotor gating impairment, and a depression-related phenotype were seen in two independent cohorts of *En2* null mutants, and in heterozygotes in some cases, as compared to wildtype littermates. Recent evidence indicates that *EN2* is a risk gene for autism [43], [44], [46]–[51]. Although the ASD-associated *EN2* rs1861972-rs1861973 A–C haplotype conveys a gain of function [45], while deletion of *En2* in mice conveys a loss of function, we detected striking social deficits in *En2* knockouts in sociability tasks that incorporate conceptual analogies to the symptoms of autism. Mice with a deletion in *En2* may represent an informative model for understanding how neurodevelopmental defects can lead to neuroanatomical or neurochemical disruptions that directly or indirectly impact behaviors relevant to psychiatric disorders.

Our behavioral findings, replicated across two cohorts of mice, demonstrate that *En2* deletion produces robust, reproducible social deficits at multiple ages and under multiple testing conditions. Both *En2* heterozygotes and null mutants displayed fewer reciprocal social interactions in freely moving dyads of same-sex juveniles and opposite-sex adult mice, specifically on parameters of investigative sniffing, following, and front approach, although not on social grooming or push-crawl behaviors. *En2* adult null mutants failed to display sociability in our three-chambered social approach task. These social deficits are qualitatively similar to those reported in mice with targeted mutations in genes implicated in autism [2], [63], [76], [90]–[92]. Our RTQPCR data indicate that *En2* is expressed in several brain structures that have been reported to mediate components of rodent social behaviors, including the hypothalamus [93]–[95], somatosensory cortex [96], hippocampus [97], striatum [93], [98] and thalamus [93]. It is interesting to speculate that deletion of *En2* in these brain structures directly or indirectly impacts social behaviors in mutant mice. Findings from our reciprocal social interaction and social approach tasks are consistent with a previous report, in which *En2* null mutants and wildtypes separately inbred as independent colonies showed reduced social interactions in freely moving pairs of sex- and genotype-matched mice [99]. The social deficits detected in *En2* mutant mice provide face validity to the aberrant social interactions and lack of interest in others that are core features of autism [100]–[102], and may be relevant to other psychiatric disorders marked by social deficits, such as schizophrenia [103]–[105].

Mutations in homeobox genes regulating early brain development often impact cognitive abilities [6], [8]–[10]. *En2* null mutant mice exhibited deficits in contextual and cued fear conditioning despite normal postshock freezing during training, deficits in acquisition of water maze hidden platform training, and lack of selective quadrant search during the probe trial. Intact hippocampal function is essential for performance on the water maze and fear conditioning [106]–[108]. A role for the cerebellum in associative fear learning has also been demonstrated [109], [110]. *En2* is expressed in multiple regions known to mediate learning and memory processes in rodents, including the hippocampus and thalamus, as detected in the present study, and the locus coeruleus and cerebellum [31], [39], [41], [99], [111]. Deletion of *En2* in these structures may produce neurobiological changes that contribute to the cognitive deficits detected in *En2* null mutant mice. Our findings of impairments in fear memory, novel object recognition memory and spatial learning may be relevant to the cognitive impairments which are frequently associated with ASD [112]–[114] and are prominent in other disorders such as schizophrenia [115]–[117].

We detected reduced PPI in two cohorts of *En2* null mutant mice, suggesting that *En2* contributes to normal sensorimotor gating. Pharmacological treatments that alter monoamine transmission disrupt PPI in rodents [118]–[120], suggesting that the PPI deficits observed in *En2* null mutants may be related to perturbations in monoamine transmitter pathways [42], [99], [111]. Prepulse inhibition deficits are an endophenotype of schizophrenia [121]–[123] and have also been reported in individuals with other disorders characterized by deficits in the gating of sensory, motor or cognitive information [121], [124], including autism [125], [126].

Forced swim and tail suspension are two tests conceptualized as “behavioral despair” paradigms, which are commonly used to detect antidepressant drug effects [127], [128]. *En2* null mutants displayed markedly higher levels of immobility on forced swim as compared to heterozygotes and wildtypes, suggesting a depression-related phenotype. Though we initially reported that male but not female null mutants displayed increased forced swim immobility [111], both sexes of null mutants displayed higher immobility times in the present study. In contrast, no genotype differences were observed in the tail suspension test, which is a putatively similar task. Changes in monoamine neurotransmitter levels differ following tail suspension versus forced swim [129], suggesting that the two tasks involve different neuronal mechanisms. Monoamine abnormalities reported in *En2* mice include reduced tyrosine hydroxylase, norepinephrine, and serotonin levels in the forebrain with increased levels of these transmitters in hindbrain structures [42], [99], [111]. Detection of a depression-related phenotype in *En2* null mutants, which replicates and extends our initial finding [111], is notable in light of reports of depression in some autistic individuals [130]–[132].

Motor functions assessed using the grip strength test and the accelerating rotarod revealed reduced forelimb grip strength in *En*2 null mutants as compared to wildtypes, and indications of rotarod deficits, consistent with previous reports [99], [133]. Detection of genotype differences in rotarod performance was dependent on the testing conditions used for each of the two cohorts. When two trials were given per day with a 1 hour intertrial interval, all three genotypes exhibited poor rotarod performance. In a second cohort given three trials per day with a 30 minute intertrial interval, which also displayed low baseline performance for all three genotypes, rotarod performance by *En2* null mutants was significantly worse than wildtypes. However, an interaction of the mutation with the background strain may be an explanatory factor for the rapid latencies to fall. 129S2/SvPas mice, the background 129 substrain originally used to generate our line of *En2* knockouts, are known to display poor performance on the rotarod and other motor tasks [134], [135]. In the present experiments, all three genotypes displayed unusually short latencies to fall, while C57BL6/J (B6) control mice displayed good rotarod performance using identical methods. However, despite the background strain phenotype, when trained with more trials at shorter intervals, *En2* null mutants showed the poorest rotarod performance of the three genotypes. These deficits are consistent with the expression of *En2* in cerebellum. The cerebellum plays a crucial role in the development of motor skills, including muscle strength [136] and motor coordination and learning [137], [138], and deletion of *En2* disrupts cerebellar development and patterning. The observed impairments in motor coordination and balance, and in neuromuscular strength, are reminiscent of the impaired motor coordination and clumsiness reported in many cases of ASD [139], [140] and offer translational read-outs of the documented anatomical abnormalities in the cerebellum of *En2* mutant mice [24]–[26], [28].

Our results demonstrate that *En2* deletion in mice reduces social behaviors on several corroborative tasks relevant to the first diagnostic symptom of autism [101], [102], [141]. Face validity for the second and third diagnostic symptoms of autism, however, was not apparent in *En2* nulls or heterozygotes. All three *En2* genotypes emitted similar numbers of ultrasonic vocalizations in social contexts as pups and adults. Similarly, responses to social olfactory cues did not differ across genotypes. Reduced ultrasonic vocalizations have been detected in other mouse models with targeted mutations in autism candidate genes [76], [77], [92], [142]–[145], as have repetitive behaviors [91], [92], [145]–[148]. Our findings suggest that *En2* mutations lead to deficits in social behaviors but not in social communication. With the exception of a trend toward increased self-grooming in the second cohort of juveniles during reciprocal social interactions, we found no evidence for increased repetitive behaviors in *En2* mutant mice. The observed lack of genotype differences is inconsistent with a previous study which reported increased self-grooming in *En2*−/− males during social interactions with a genotype- and sex-matched partner [99]. Differences in testing or housing conditions might have contributed to the divergent findings.

No genotype differences were detected for parameters of neurobehavioral development, general health, pain sensitivity, open field locomotor activity, anxiety-like behaviors, sensory abilities, acoustic startle reactivity and pain sensitivity, with the exception of small differences in some measures of light ↔ dark exploration and open field activity. Findings from these control tasks indicate that the social abnormalities detected in *En2* mutant mice cannot be attributed to an obvious physical defect or confounding phenotype. Our findings indicate a specific social deficit in *En2* null mutants, recapitulating the first diagnostic symptom of autism, without abnormalities in the communication and repetitive symptom domains.

Our RTQPCR results recapitulate previous findings demonstrating that *En2* is expressed in the hippocampus and cortex in wild type but not *En2*−/− mice [39]. We have now extended this analysis by showing that *En2* is transcribed at high or intermediate levels in cerebellum, colliculi, brainstem and thalamus, while low level expression is seen in the hypothalamus, hippocampus, striatum and somatosensory cortex. These results demonstrate that *En2* is widely expressed in adult brain structures, some of which contribute to behaviors relevant to autism and other psychiatric disorders. For example, social behaviors in rodents are regulated in part by the hypothalamus [94], and cognitive and sensorimotor abilities are regulated in part by the hippocampus [108], [149], [150], somatosensory cortex [96], [151] thalamus [152]–[154], striatum [98], [125], [154], [155] and brainstem [153], [156]. No expression of *En2* was detectable in *En2−/−* mice, as expected. Lack of *En2* expression in these adult structures could contribute directly or indirectly to the behavioral abnormalities observed in the *En2*−/− mice. Further, the *En2* mutation results in numerous aberrations in brain development, including connectivity defects, which could contribute to the behavioral phenotypes. En2 protein has been detected in both the nucleus and in vesicles of neurons, and a small proportion of the protein is secreted [157], [158]. Investigations of subcellular localization of En2 within forebrain regions might shed light on its role in development of brain structures responsible for complex behaviors relevant to autism and other disorders.

*En2* heterozygotes generally resembled wildtypes, although trends for intermediate phenotypes appeared on selected parameters of juvenile reciprocal social interactions, Morris water maze spatial learning, forelimb grip strength, and rotarod motor coordination and balance, indicating the possibility of gene dose effects. Consistent with a previous report [99], we did not detect sex differences in any of the behavioral abnormalities observed in *En2* null mutants. The occurrence of autism is significantly higher in males than in females, with a male to female ratio of 4∶1 [159]. Thus, an animal model that displays relevant phenotypes in males but not females could be considered to have face validity with regard to the prevalence of ASD. Sex differences have been reported for a few mouse models of autism. For example, social deficits have been detected in male but not female mice of the inbred C58/J strain [160], and male mice with a deletion in *Shank3* display more severe impairments in motor coordination as compared to females [63], [148]. However, the majority of studies have detected autism-relevant behavioral phenotypes in both male and female mice (e.g. [77], [146], [161]–[163]). It is possible that mutations in mice are not as likely to result in sex-specific differences that appear in humans, for mechanistic reasons that will be interesting to explore.

Our comprehensive behavioral findings, which appear remarkably similar across two independent cohorts of wildtype, heterozygous and null mutant *En2* mice, confirm a previous report of reduced social interactions in *En2* null mutants bred separately from wildtypes [99], confirming the robustness and replicability of these behavioral abnormalities in *En2* mice, independent of breeding strategy. We further replicated our initial finding of a depression-relevant phenotype, which provides a functional read-out relevant to monoamine abnormalities in *En2* null mutant mice [111]. The neuroanatomical expression pattern of *En2* found in our wildtype and mutant mice supports mechanistic hypotheses about anatomical disruptions in brain regions mediating social behaviors, cognitive abilities, depression-relevant behaviors, sensorimotor gating, and motor functions [108], [124], [164]–[166]. Our results suggest new directions for understanding the precise role of *EN2* in elaborating neuroanatomical circuits during early brain development, which may contribute the symptoms of autism and other neurodevelopmental and psychiatric disorders.
